# Hyperproduction of a Fungal Natural Product, Calcarisarbulide A, From a Synthetic Transcriptional Cascade

**DOI:** 10.3390/jof12100742

**Published:** 2026-10-03

**Authors:** Ji-Jin Xie, Yi Wang, Chun-Feng Wang, Han Wang, Fan Xu, Bei-Bei Wang, Daniel H. Scharf, Xu-Ming Mao

**Affiliations:** 1 Department of Clinical Pharmacy, The First Affiliated Hospital, School of Medicine, Zhejiang University, Hangzhou 310058, China; 2 Institute of Pharmaceutical Biotechnology, School of Medicine, Zhejiang University, Hangzhou 310058, China; 3 Zhejiang Provincial Key Laboratory of Synthetic Biotechnology for Microbial Medicine, Hangzhou 310058, China; 4 Polytechnic Institute, Zhejiang University, Hangzhou 310015, China

**Keywords:** fungal natural product, hyper production, synthetic biology, transcriptional cascade

## Abstract

Effective hyperproduction of fungal natural products (NPs) is one of the challenges in their pharmaceutical development. Calcarisarbulide A (**1**) is a promising anti-liver-fibrosis NP from the endophyte *Calcarisporium arbuscula*. Here, based on a hybrid transcription factor used to awaken the *cal* gene cluster (GC) in *C. arbuscula* and genetic engineering to block the biotransformation of **1**, a yield of 1.13 g/L was obtained. To produce **1** more safely and at higher yields, heterologous expression in *Aspergillus* was demonstrated. However, the yields of **1** were much lower in *A. nidulans* and *A. terreus*, likely due to the endogenous metabolism of **1**. In *A. oryzae*, where **1** is not metabolized, a synthetic transcriptional cascade was developed from the *A. nidulans afo* GC to produce 1.31 g/L of **1**. Further aspartic acid feeding led to a 2.62 g/L yield. Our work provides a new synthetic biology strategy for the efficient production of bioactive fungal NPs.

## 1. Introduction

Filamentous fungi are a rich source of bioactive natural products (NPs) for pharmaceutical development [1]. Genome sequencing projects further revealed the vast potential of fungal gene clusters (GCs) to produce NPs, while most of them are untapped [2]. However, most fungal NPs with promising prospects are produced in low yields, which dramatically impedes their further development [3,4].

*Aspergillus oryzae* and *Aspergillus nidulans* have been widely used as fungal chassis for heterologous production of NPs [3,4]. In particular, *A. oryzae*, with a long history of safe use in the production of fermented foods and food enzymes that have received GRAS status, has been widely used for biosynthetic research [5]. In this fungus, the strong *amyB* promoter has been adopted to express biosynthetic enzymes for NP production, though mostly at low yields [3]. However, in *A. nidulans*, promoters and the pathway-specific regulator AfoA from the *afo* GC have been used as synthetic transcriptional elements to construct a transcriptional cascade and drive more efficient production of NPs than systems in which biosynthetic enzymes are expressed directly under strong promoters [6]. These studies imply that the build-up of effective transcriptional cascades may represent a more promising approach for the hyperproduction of fungal NPs.

With increasing life expectancy and the prevalence of chronic liver diseases, liver fibrosis is becoming one of the major challenges of global health. If left untreated, it will progress to life-threatening cirrhosis and hepatocellular carcinoma. However, there are currently no approved pharmaceuticals for liver fibrosis [7,8]. Calcarisarbulide A (**1**), formerly named MCA17-1, is produced by the mushroom endophyte *Calcarisporium arbuscula* and exhibits remarkable activity in suppressing TGF-β-induced differentiation of hepatic stellate cells, suggesting that **1** is a promising NP for anti-liver-fibrosis development [9]. However, the yield of **1** remains unsatisfactorily low.

Here, a new *C. arbuscula* strain was developed through multiple genetic engineering modifications to produce **1** at 1.13 g/L. Moreover, **1** was produced in heterologous hosts including *A. nidulans* and a lovastatin-producing *A. terreus* strain, though at low yields, likely due to the inherent metabolism of **1** in these fungi. We further developed an *A. oryzae* strain, which does not metabolize **1,** for the hyperproduction of **1** to 2.62 g/L based on exogenous synthetic transcriptional cascades and precursor feeding. This work provides a new platform with a combination of fungal hosts and genetic elements for hyperproduction of bioactive NPs, and also shows that non-inherent metabolism of NPs is one of the critical factors for their effective biosynthesis.

## 2. Materials and Methods

### 2.1. Bacterial and Fungal Strains

The bacterial and fungal strains are listed in Table S1. In brief, *Escherichia coli* DH5α (Tsingke Biotechnology Co., Ltd., Beijing, China) was used for subcloning, while *Agrobacterium tumefaciens* EHA 105 was used as a host for *Agrobacterium tumefaciens*-mediated transformation (ATMT) to transfer DNA from bacteria to *C. arbuscula* [10]. 

*C. arbuscula* NRRL 3705 [10] is an endophyte containing the *cal* GC. *Aspergillus nidulans* LO8030 [11], *Aspergillus oryzae* with *pyrG* auxotroph (RIB40 origin) [12], and *Aspergillus terreus* XM381 are the hosts for the production of **1**. The Whole Genome Shotgun project of *A. terreus* XM381 has been deposited in DDBJ/ENA/GenBank under the accession number JCAQIV000000000. The version described in this paper is version JCAQIV010000000.

### 2.2. Cell Growth Conditions and Strain Development

Growth of *E. coli* for subcloning, *A. tumefaciens* for ATMT, and *C. arbuscula* for genetic engineering has been described previously [10]. Cultivation of *A. nidulans* and *A. oryzae* was performed as previously described [3,4,6]. *A. terreus* was routinely grown on potato dextrose agar (BD Difco, Franklin Lakes, NJ, USA) at 37 °C for spore preparation. All *A. terreus* mutants were developed based on CRISPR-Cas9 genome editing [13]. Details of genetic manipulation are described in the Supplementary Information. The CD (Czapek-Dox) medium (BD Difco, Franklin Lakes, NJ, USA) used in this study contains 30 g/L sucrose, 3 g/L NaNO_3_, 1 g/L KH_2_PO_4_, 0.5 g/L MgSO_4_·7H_2_O, 0.5 g/L KCl, and 0.01 g/L FeSO_4_.

Genetic manipulation strategies: three distinct expression strategies were employed in this study depending on the host and the desired genetic outcome. Genome integration via homologous recombination was used in *C. arbuscula* (ATMT-mediated) and *A. terreus* (CRISPR-Cas9-mediated) at the *aurA* and *lov* loci, respectively. Plasmid-based extrachromosomal expression was used in *A. nidulans* and *A. oryzae* using AMA1-based replicative vectors (pFC902; laboratory stock, Zhejiang University, Hangzhou, China).

All genome-integrated strains were confirmed by diagnostic PCR using primers flanking the integration sites and primers specific to the integrated cassettes (all primers synthesized by Tsingke Biotechnology Co., Ltd., Beijing, China); the PCR products were further verified by Sanger sequencing (Tsingke Biotechnology Co., Ltd., Beijing, China). For AMA1-based plasmid-containing strains, plasmid maintenance was confirmed by PCR amplification using vector- and cassette-specific primers, as well as by phenotypic selection on media lacking uracil/uridine (for the *pyrG* marker).

### 2.3. Plasmid Development

All plasmids and primers used in this study are listed in Tables S2 and S3, respectively. The procedures for all subcloning are described in the Supplementary Information.

### 2.4. Chemical Analysis

Compounds **2**–**4** were purified from the cultures of *C. arbuscula* and characterized as described in the Supplementary Information. Crude extract preparation, HPLC (Shimadzu LC-20ADXR, Shimadzu, Kyoto, Japan), and HR-MS (AB SCIEX ZENO Q-TOF 7600, AB SCIEX, Framingham, MA, USA) analysis are also described in the Supplementary Information. Data were processed using LabSolutions version 5.99 (Shimadzu, Kyoto, Japan) and Analyst TF version 1.7.1 (AB SCIEX, Framingham, MA, USA).

### 2.5. Measurement of Fungal Biomass (Pmv)

To monitor the fungal growth kinetics, the Packed Mycelial Volume (PMV) was measured daily. Briefly, a defined volume of the fermentation culture was sampled into a graduated centrifuge tube (NEST Biotechnology Co., Ltd., Wuxi, Jiangsu, China) and centrifuged at 13,000 rpm for 10 min using an Eppendorf 5424R centrifuge (Eppendorf, Hamburg, Germany). The volume of the clear supernatant was carefully recorded. The PMV was calculated by subtracting the ratio of the supernatant volume from the total sample volume, expressed as a percentage (*v*/*v*, %).

### 2.6. Statistics and Reproducibility

All data are presented as the mean ± SD from at least three independent experiments. Statistical significance was determined using an unpaired two-tailed Student’s *t*-test for two-group comparisons using GraphPad Prism version 8.0.2 (GraphPad Software, San Diego, CA, USA). A *p*-value < 0.05 was considered statistically significant.

## 3. Results

### 3.1. Development of a C. arbuscula Strain with a High Yield of ***1*** by Multiple Target Genetic Engineering

**Figure 1:**
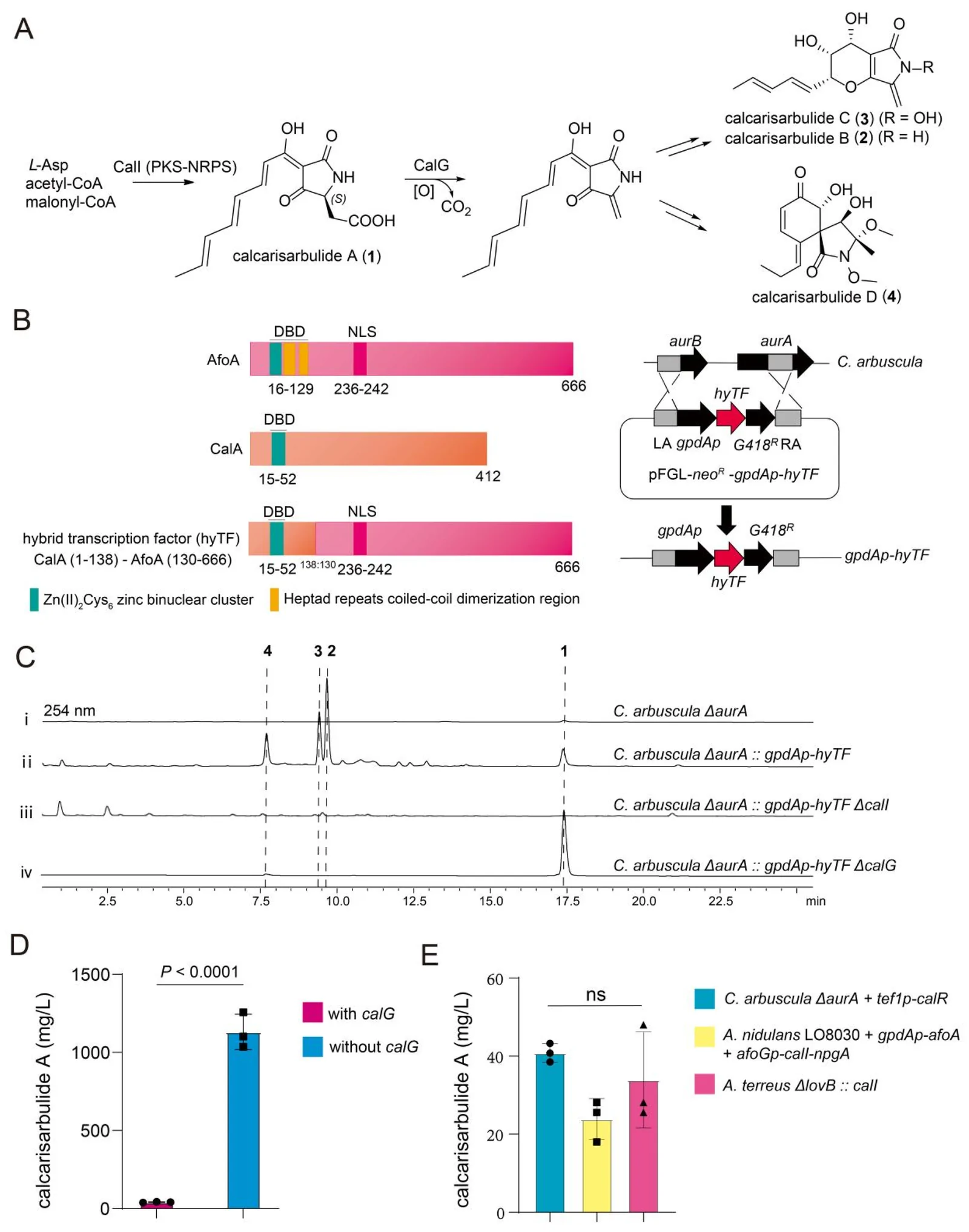
Genetic engineering to improve production of **1** in *C. arbuscula*. (**A**) Chemical structures of compounds **1**–**4** from *C. arbuscula* and their proposed biosynthetic pathway. **1**, calcarisarlrubide A; **2**, calcarisarlrubide B (R = H); **3**, calcarisarlrubide C (R = OH); **4**, calcarisarlrubide D. (**B**) The genetic strategy to activate *cal* GC in *C. arbuscula* by overexpression of a hybrid transcription factor at the *aurA* locus. (**C**) HPLC analysis of *C. arbuscula* strains with different genetic backgrounds. (**D**) The yields of **1** in *C. arbuscula* with (magenta bars) or without (blue bars) *calG* in PDB after cultivation for 2 weeks. Data are presented as mean ± SD from three independent experiments. *p* < 0.0001 (two-tailed Student’s *t*-test). (**E**) The yield of compound **1** in *A. nidulans* LO8030 harboring the *afoGp-calI* + *gpdAp-afoA* plasmid (yellow bar) and *A. terreus ΔlovB::calI* (pink bar), compared with *C. arbuscula ΔaurA* + *tef1p-calR* (blue bar). Cultures were grown in PDB for 14 days. ns, not significant (two-tailed Student’s *t*-test). Data are presented as mean ± SD from three independent experiments.

In *C. arbuscula*, **1** is biosynthesized by a polyketide synthase–non-ribosomal peptide synthetase (PKS-NRPS) hybrid enzyme in the *cal* GC (formerly named *mca17*) (Figure 1A) [9]. This GC contains 14 genes, including a core enzyme gene *calI* encoding PKS-NRPS and a regulatory gene *calR* encoding a pathway-specific transcription factor. Previous work involving random integration of *calR* under *tef1p* awakened this cryptic GC to produce 34.48 mg/L **1** [9]. To produce **1** in a higher yield in this native producer, we developed a hybrid transcription factor, CalRA, by fusion of the N-terminal DNA-binding domain of CalR to the C-terminal activation domain of AfoA from *A. nidulans* [14]. Moreover, since *C. arbuscula* predominantly produces mycotoxins aurovertins from the *aur* GC, our hybrid transcription factor, expressed under a stronger promoter *gpdAp* [10], was integrated at the *aurA* locus, which is transcriptionally active and would be a hotspot for ectopic expression [15,16] (Figure 1B). This strategy led to prominent production of **1** (approximately 40.87 mg/L) and three new NPs, calcarisarbulide B–D (**2**–**4**), with intriguing ring structures, compared to the *ΔaurA* mutant [17] (Figure 1A–C). **2**–**4** are proposed to be derived from **1**, as deletion of *calI* led to no production of **1**–**4** (Figure 1C). The structures and the stereochemistry of **2**–**4** were determined based on HR-MS, NMR, and ECD (Figures S1–S27, Tables S4–S9).

To increase **1** production, we deleted *calG*, which is proposed to encode an oxidative decarboxylase [9] that catalyzes the first step of post-assembly tailoring. In this *ΔcalG* mutant, we only observed **1** without production of **2**–**4** (Figure 1C). Further flask fermentation showed a yield of about 1.13 g/L of **1** from this mutant at day 14, while the *calG* intact strain produced only about 40.87 mg/L of **1** (Figure 1D).

### 3.2. Heterologous Biosynthesis of ***1*** in A. nidulans and A. terreus

*C. arbuscula* is an endophyte that sporulates poorly and grows optimally at 25 °C. Moreover, genome sequencing suggests that it potentially produces some mycotoxins, including aurovertins [15,17]. These disadvantages impede its further development as a producer of **1** for anti-liver-fibrosis pharmaceuticals. Thus, we turned to heterologous production of **1** in *Aspergillus*.

*A. nidulans* is a commonly used host for NP production [4], and high yields of NPs can be obtained if exogenous GCs are placed at the *afo* locus and driven from the *afo* promoters, along with overexpression of the GC-specific transcription factor AfoA [6]. We expressed *calI* under the *afoG* promoter (*AN01036*), a PKS gene for asperfuranone biosynthesis, and expressed *afoA* (*AN01026*) from *gpdAp* on an AMA1-based high-copy vector. However, we only obtained about 23.89 mg/L **1** in *A. nidulans* LO8030 (cultured in PDB for 14 days, Figure 1E), where eight active biosynthetic GCs have been eliminated [11].

We initially speculated that this unsatisfactory yield of **1** in *A. nidulans* might result from incompatibility between the *cal* GC and *afo* promoters or from *calI* not being placed at a transcriptionally active locus (i.e., the *afo* locus) [6]. To investigate whether the poor production of **1** in *A. nidulans* was due to the specific genomic context (i.e., the *afo* GC) or the fungal host itself, we next explored a different transcriptionally active locus in a lovastatin-overproducing *A. terreus* strain. We have an in-house *A. terreus* strain XM381 that produces the PKS-type NP lovastatin at a high yield of about 3.78 g/L in laboratory PDB culture (Figure S28), suggesting that the *lov* locus would be a promising hotspot for heterologous expression [16]. This *lov* GC was readily obtained after whole-genome sequencing, and the PKS gene *lovB* was replaced with *calI* by CRISPR-Cas9-based genome editing. However, this strategy yielded only about 33.91 mg/L **1**, even lower than the original yield from *C. arbuscula* (cultured in PDB for 14 days, Figure 1E). This result is consistent with the results from *A. nidulans*, but is inconsistent with the high lovastatin yield in *A. terreus*. 

**Figure 2:**
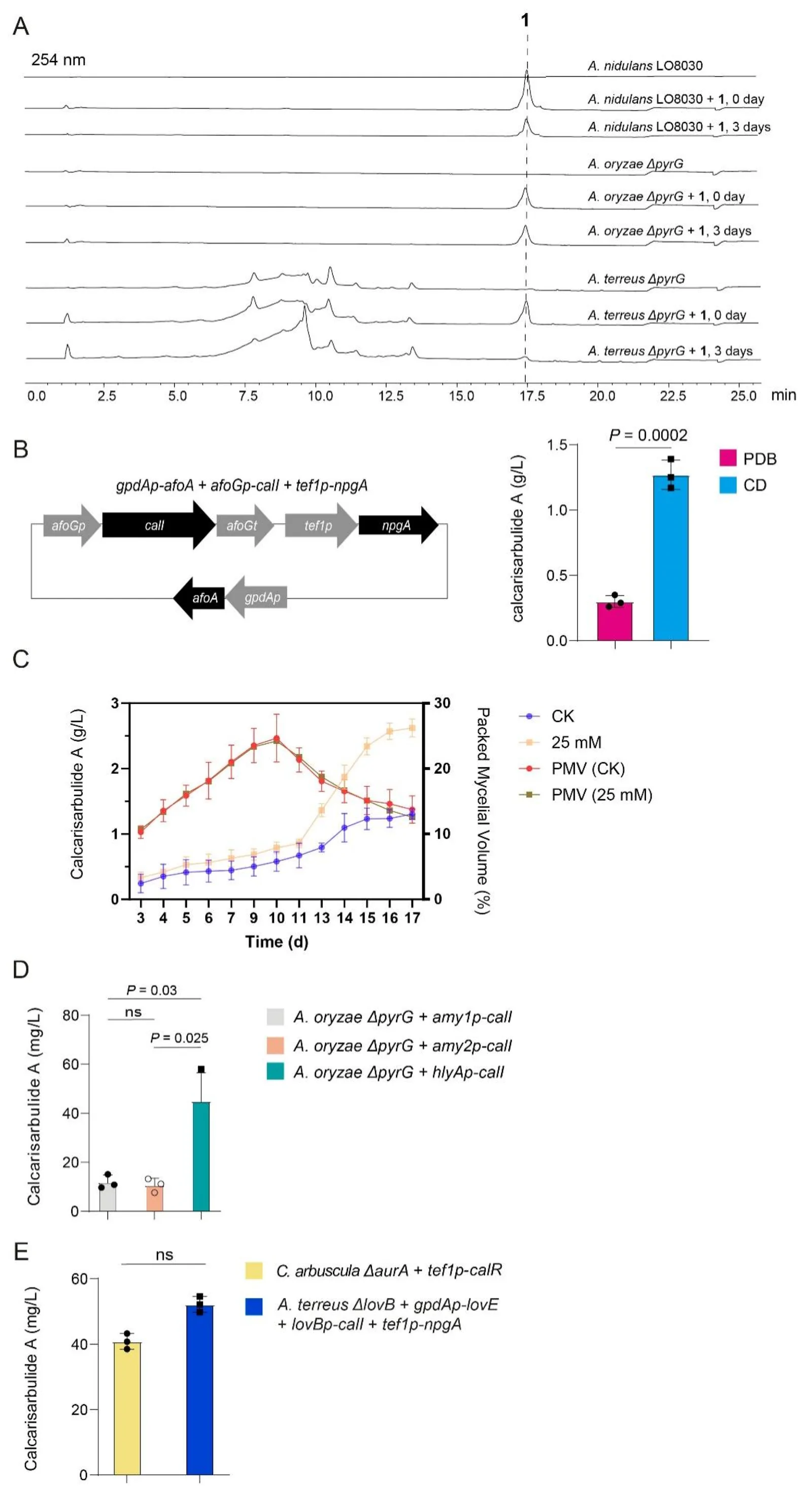
Heterologous production of **1** in *A. oryzae*. (**A**) Feeding assays of **1** in *A. nidulans* LO8030, *A. terreus* XM381, and *A. oryzae* RIB40 in a *ΔpyrG* background. After culturing *Aspergillus* species in PDB for 3 days, about 50 μg of **1** was fed, and samples were collected on day 0 and day 3 after feeding of **1**. (**B**) Hyperproduction of **1** from an *afo*-based synthetic transcriptional cascade in *A. oryzae*. *calI* is expressed under *afoGp*, along with *afoA* and *npgA* expressed from *gpdAp* and *tef1p*, respectively. **1** was produced from the above expression cassette after cultivation in PDB (magenta bar) and CD (blue bar), respectively. The yields of **1** were monitored on day 14. (**C**) Enhanced production of **1** and biomass monitoring in *A. oryzae* with 25 mM Asp supplementation in CD medium. The yields of **1** (circles) and packed mycelial volume (PMV; squares) were examined from day 3 to day 17 in flask fermentation. CK, control (blue circles for **1**; red squares for PMV); 25 mM Asp (orange circles for **1**; green squares for PMV). (**D**) Production of compound **1** from the *amy*-activation system in *A. oryzae* strains. *calI* was expressed under *amy1p* (grey bar), *amy2p* (orange bar), or *hlyAp* (teal bar) promoters in 2% flour medium for 14 days. Data are presented as mean ± SD from three independent experiments. ns, not significant; *p* = 0.03 and *p* = 0.025 (two-tailed Student’s *t*-test). (**E**) Production of compound **1** from the *lov*-based synthetic transcriptional cascade in *A. oryzae*. Cultures were grown in CD medium for 14 days. Yellow bar, *C. arbuscula* Δ*aurA* + *tef1p-calR*; blue bar, *A. terreus* Δ*lovB* + *gpdAp-lovE* + *lovBp-calI* + *tef1p-npgA*. Data are presented as mean ± SD from three independent experiments. ns, not significant (two-tailed Student’s *t*-test).

To investigate whether the low yields of compound **1** in *A. nidulans* and *A. terreus* were due to endogenous metabolism, we performed feeding assays in *A. nidulans* LO8030, *A. terreus* XM381, and *A. oryzae* RIB40. As shown in Figure 2A, compound **1** disappeared after three days in both *A. nidulans* and *A. terreus*, but remained stable in *A. oryzae* throughout the examined period. These results suggest that *A. oryzae*, which does not metabolize compound **1**, would be a more suitable chassis for high-yield production. Notably, no compound **1** was detected in the parental strains *A. nidulans* LO8030 or *A. terreus* XM381 (Figure 1E), confirming that the low but detectable yields observed in the engineered strains are derived from the heterologously expressed *calI* rather than endogenous production. BLAST 2.2.26 (National Center for Biotechnology Information, Bethesda, MD, USA) analysis against the genomes of both hosts revealed that while homologs of the core PKS-NRPS gene *calI* are present, a complete *cal* GC is absent, consistent with the lack of endogenous production in the parental strains (Table S10).

### 3.3. Production of ***1*** in A. oryzae with Synthetic Transcriptional Cascades

To evaluate heterologous production of **1**, we employed two distinct expression strategies: chromosomal integration via CRISPR-Cas9 in *A. terreus* (at the *lov* locus) and AMA1-based plasmid expression in *A. nidulans* (using the *afo* transcriptional cascade system). 

*A. oryzae* is renowned for its secreted amylase for starch degradation. Initially, we put *calI* on a multiple-copy vector under the promoters of *amy1* (*AO090023000944*) or *amy2* (*AO090120000196*), as well as *hlyA* (*AO090010000016*), which have been used as starch-inducible strong promoters for NP production in *A. oryzae* [3]. However, these endogenous transcriptional elements only resulted in about 11.87 mg/L, 10.67 mg/L, and 44.96 mg/L **1**, respectively, in the flour-containing medium used to induce the expression of these promoters (Figure 2D).

Next, we developed a transcriptional cascade in *A. oryzae* based on exogenous transcriptional elements from the *A. terreus lov* and *A. nidulans afo* GCs. The *calI* gene was expressed under the promoters of *lovB* and *afoG* (both of which encode PKS), respectively, on the replicative vector, while the GC-specific transcriptional factors LovE and AfoA were expressed under the constitutive strong promoter, *gpdAp,* from *A. nidulans*, along with *A. nidulans npgA* (*AN06140*) (encoding a 4′-phosphopantetheine transferase) expressed from the *A. nidulans tef1* promoter to potentially improve the catalytic activity of PKS-NRPS CalI.

Though lovastatin is predominantly produced in *A. terreus* at 3.78 g/L, this synthetic expression cascade *gpdAp*-*lovE* + *lovBp-calI* only resulted in 52.20 mg/L **1** in the synthetic CD medium (Figure 2E). However, when the synthetic transcriptional cascade *gpdAp*-*afoA* + *afoGp-calI* was expressed in *A. oryzae*, we obtained 0.30 g/L **1** in PDB, but intriguingly 1.27 g/L **1** in CD (Figure 2B). These data suggested that the *afo*-based synthetic transcriptional cascade was more compatible with *A. oryzae*, most probably due to the more efficient transcriptional activation of the regulator in this fungal host (i.e., herein AfoA versus LovE). We proposed that the higher yield in CD was likely attributable to plasmid stability—CD provides continuous selection for the *pyrG* marker on the AMA1 plasmid, while PDB lacks this selection and allows plasmid loss.

Given that **1** was biosynthesized from aspartic acid (Asp) and acetyl-CoA as the precursors, we speculated that adequate precursors, particularly Asp, were required for further improvement of **1** production. A single dose of 25 mM aspartic acid was added to the CD medium on day three, not daily. Our Asp-feeding fermentation showed that no yield difference was observed before day 11, when **1** reached a yield of 0.85 g/L, and finally 1.31 g/L yield was obtained. However, a high concentration of Asp (25 mM) was beneficial in boosting **1** production after day 11, and the yield eventually reached 2.62 g/L on day 17. Importantly, packed mycelial volume (PMV) analysis confirmed that no secondary growth occurred after day 11, indicating that this late-stage yield boost was driven by sustained biosynthesis rather than cell proliferation (Figure 2C). It is proposed that *A. oryzae* has sufficient Asp supply from endogenous biogenesis and the medium to produce **1** at the early stage, but cannot support further hyperproduction of **1** without Asp feeding. These data provide a basis for further optimization of the scale-up production of **1**.

## 4. Discussion

Fungal NPs have been important sources of microbial pharmaceuticals for human health and agriculture, as well as many additives for daily life [1]. However, though great efforts have been made in the discovery, biosynthesis, and engineering of fungal NPs, the yields of most fungal NPs are too low for industrial applications [4], except for a few well-established products (such as penicillin and lovastatin). Thus, it is highly urgent to develop orthogonal expression systems for hyperproduction of fungal NPs, which mainly include appropriate fungal chassis and transcriptional elements.

Many environment-derived filamentous fungi have biosynthetic gene clusters (BGCs) to produce biohazardous mycotoxins, which might be beneficial for their survival and competition [18]. Thus, it is desirable to produce fungal NPs in a safe host to prevent possible mycotoxin contamination. While some widely used *Aspergillus* species also produce mycotoxins, for example, sterigmatocystin from *A. nidulans* [19] and citreoviridin from *A. terreus* [20], *A. oryzae*, a species with a long history of producing GRAS-certified ingredients, has been proven safe in the fermentation industry. Another important consideration is that fungal NPs should not be enzymatically metabolized by the heterologous host, as many fungal species have homologous biosynthetic GCs or isoenzymes to convert target NPs to other compounds. For example, we have previously reported biotransformation of structurally related mycotoxins in fungal genera, though it is also beneficial for structural diversity [21]. Here, among several *Aspergillus* species, **1** is stable only in *A. oryzae*, which makes this species an ideal chassis for hyperproduction of fungal NPs.

*A. oryzae* produces abundant amylases for starch degradation. Though the transcriptional elements from the amylase system have been used to drive production of NPs in *A. oryzae*, here we showed that they are not strong enough to produce NPs in high yields. Instead, the expression system from the *A. nidulans afo* GC is more compatible with driving hyperproduction of NPs, such as calcarisarbulide A (**1**) in this study, making this orthogonal expression system a promising platform for other fungal NPs. Moreover, there are eight genes within this *afo* GC under the positive regulation of AfoA [6], suggesting that promoters from the *afo* GC can also be used to express complex fungal GCs without repetitive usage of strong promoters in *A. oryzae*. In addition, several neutral integration sites with active transcription have been identified in the *A. oryzae* genome [16]. These hotspot sites further provide opportunities for stable expression of multiple copies of fungal GCs, leading to hyperproduction of bioactive fungal NPs. While compound **1** has shown promising anti-liver-fibrosis activity without obvious cytotoxicity at effective concentrations, a comprehensive toxicological evaluation remains to be established in future studies.

## 5. Conclusions

There is a lack of efficient orthogonal expression systems for hyperproduction of fungal NPs. Here, based on a hybrid transcription factor and targeted genetic engineering, a high-yield strain of **1** has been developed in its native producer, *C. arbuscula*. For safer and more efficient production of **1**, several *Aspergillus* species were examined for heterologous expression. We showed that the lack of endogenous metabolism of **1** is critical for high-level heterologous production in *Aspergillus*. Such an *A. oryzae* strain was developed based on a synthetic transcriptional cascade from *A. nidulans afo* GC to produce **1** at a yield of 2.62 g/L. Our work provides a new platform for hyperproduction of bioactive fungal NPs.

## Data Availability

The raw data supporting the conclusions of this article will be made available by the authors on request.

## Supplementary Materials

The following supporting information can be downloaded at: https://www.mdpi.com/article/10.3390/jof12100742/s1, Table S1: All strains used in this study; Table S2: Plasmids used in this study; Table S3: All primers used in this study; Table S4: The chemical shift of compound **2** in CD_3_OD; Table S5: The chemical shift of compound **3** in CD_3_OD; Table S6: The chemical shift of compound **4** in CDCl_3_; Table S7: Energy analysis for (8R,9R,10R,11S)-**4** and (6*S*,7*S*,8*S*)-**2**; Table S8: Calculated ECD data for (8*R*,9*R*,10*R*,11*S*)-**4**; Table S9: Calculated ECD data for (6*S*,7*S*,8*S*)-**2**; Table S10: BLAST analysis of *cal* GC homologs in *A. nidulans* LO8030 and *A. terreus* XM381; Figure S1: HRMS and UV spectra of compound **2**; Figure S2: ^1^H NMR of compound **2** in CD_3_OD at 600 MHz; Figure S3: ^13^C NMR of compound **2** in CD_3_OD at 150 MHz; Figure S4: DEPT135 NMR of compound **2** in CD_3_OD at 150 MHz; Figure S5: ^1^H-^1^H COSY of compound **2** in CD_3_OD; Figure S6: HSQC of compound **2** in CD_3_OD; Figure S7: HMBC of compound **2** in CD_3_OD; Figure S8: NOESY of compound **2** in CD_3_OD; Figure S9: HRMS and UV spectra of compound **3**; Figure S10: ^1^H NMR of compound **3** in CD_3_OD at 600 MHz; Figure S11: ^13^C NMR of compound **3** in CD_3_OD at 600 MHz; Figure S12: DEPT135 of compound **3** in CD_3_OD at 150 MHz; Figure S13: ^1^H-^1^H COSY of compound **3** in CD_3_OD; Figure S14: HSQC of compound **3** in CD_3_OD; Figure S15: HMBC of compound **3** in CD_3_OD; Figure S16: HRMS and UV spectra of compound **4**; Figure S17: ^1^H NMR of compound **4** in CDCl_3_ at 600 MHz; Figure S18: ^13^C NMR of compound **4** in CDCl_3_ at 150 MHz; Figure S19: ^1^H-^1^H COSY of compound **4** in CDCl_3_; Figure S20: HSQC of compound **4** in CDCl_3_; Figure S21: HMBC of compound **4** in CDCl_3_; Figure S22: NOESY of compound **4** in CDCl_3_; Figure S23: Comparative analysis of CD spectra between compound **2** and spirostaphelotrichin X; Figure S24: Experimental ECD curves of **4**; Figure S25: Experimental ECD curves of **2**; Figure S26: B3LYP/6-31G(d) optimized lowest energy conformers for (8*R*,9*R*,10*R*,11*S*)-**4**; Figure S27: B3LYP/6-31G(d) optimized lowest energy conformers for (6*S*,7S,8S)-**2**; Figure S28: HPLC analysis of *A. terreus* and identification of lovastatin. Refs [6,10,17,22,23,24,25,26] are cited in Supplementary Materials file.
