# Supplementary material for: Hyperproduction of a Fungal Natural Product, Calcarisarbulide A, From a Synthetic Transcriptional Cascade
Source: J Fungi (Basel). 2026 Oct 3;12(10):742. doi: 10.3390/jof12100742 (PMC13705927; doi:10.3390/jof12100742)
Supplement: Supplementary file 1 [file jof-12-00742-s001.zip › jof-4525836-supplementary.pdf]

Supplementary Information

Hyper-production of a fungal natural product calcarisarbulide A from a synthetic transcriptional cascade

Ji-Jin Xie[#1,3,4], Yi Wang[#1,2,3,4], Chun-Feng Wang[1,2,3,4], Han Wang[3,4], Fan Xu[3], Bei-Bei Wang[1,3,4], Daniel H. Scharf[3], Xu-Ming Mao*[1,3,4]

[1]Department of Clinical Pharmacy, the First Affiliated Hospital, School of Medicine, Zhejiang University, Hangzhou 310058, P.R.China

[2]Polytechnic Institute, Zhejiang University, Hangzhou 310015, P.R.China

[3]Institute of Pharmaceutical Biotechnology, School of Medicine, Zhejiang University, Hangzhou 310058, P.R.China

[4]Zhejiang Provincial Key Laboratory of Synthetic Biotechnology for Microbial Medicine, Hangzhou 310058, P.R.China

[#]These authors contributed equally to this work.

*Correspondence: Prof. Xu-Ming Mao: xmmao@zju.edu.cn

Table of Contents

Supplementary Information 1
Methods 1
Bacterial strains, culture conditions, plasmids and reagents 1
Strain development 1
Plasmid construction 2
Compound purification 3
Structural determination of compounds **2**–**4** 4
Table S1. All strains used in this study. 6
Table S2. Plasmids used in this study. 7
Table S3. All primers used in this study. 8
Table S4. The chemical shift of compound **2** in CD_3OD. 10
Table S5. The chemical shift of compound **3** in CD_3OD. 11
Table S6. The chemical shift of compound **4** in $CDCl_3$. 12
Table S7. Energy analysis for (8*R*,9*R*,10*R*,11*S*)-**4** and (6*S*,7*S*,8*S*)-**2**. 13
Table S8. Calculated ECD Data for (8*R*,9*R*,10*R*,11*S*)-**4**. 14
Table S9. Calculated ECD Data for (6*S*,7*S*,8*S*)-**2**. 17
Table S10. BLAST analysis of cal GC homologs in *A. nidulans* LO8030 and *A. terreus* XM381 17
Figure S1. HRMS and UV spectra of compound **2**. 20
Figure S2. ^{1}H NMR of compound **2** in CD_3OD at 600 MHz. 21
Figure S3. ^{13}C NMR of compound **2** in CD_3OD at 150 MHz. 22
Figure S4. DEPT135 NMR of compound **2** in CD_3OD at 150 MHz. 23
Figure S5. ^{1}H-^{1}H COSY of compound **2** in CD_3OD. 24
Figure S6. HSQC of compound **2** in CD_3OD. 25
Figure S7. HMBC of compound **2** in CD_3OD. 26
Figure S8. NOESY of compound **2** in CD_3OD. 27
Figure S9. HRMS and UV spectra of compound **3**. 28
Figure S10. ^{1}H NMR of compound **3** in CD_3OD at 600 MHz. 29
Figure S11. ^{13}C NMR of compound **3** in CD_3OD at 600 MHz. 30

Figure S12. DEPT135 of compound **3** in CD_3OD at 150 MHz. 31
Figure S13. ^{1}H-^{1}H COSY of compound **3** in CD_3OD. 32
Figure S14. HSQC of compound **3** in CD_3OD. 33
Figure S15. HMBC of compound **3** in CD_3OD. 34
Figure S16. HRMS and UV spectra of compound **4**. 35
Figure S17. ^{1}H NMR of compound **4** in $CDCl_3$ at 600 MHz 36
Figure S18. ^{13}C NMR of compound **4** in $CDCl_3$ at 150 MHz. 37
Figure S19. ^{1}H-^{1}H COSY of compound **4** in $CDCl_3$. 38
Figure S20. HSQC of compound **4** in $CDCl_3$. 39
Figure S21. HMBC of compound **4** in $CDCl_3$. 40
Figure S22. NOESY of compound **4** in $CDCl_3$. 41
Figure S23. Comparative analysis of CD spectra between compound **2** and Spirostaphylotrichin X 42
Figure S24. Experimental ECD curves of **4** (solid black line), M062X/TZVP//B3LYP/6-31G(d) calculated ECD spectra of (8*R*,9*R*,10*R*,11*S*)-**4** (solid red line) and (8*S*,9*S*,10*S*,11*R*)-4 (dash red line). 43
Figure S25. Experimental ECD curves of **2** (solid black line), M062X/TZVP//B3LYP/6-31G(d) calculated ECD spectra of (6*R*,7*R*,8*R*)-2 (solid red line) and (6*S*,7*S*,8*S*)-2 (dash red line). 44
Figure S26. B3LYP/6-31G(d) optimized lowest energy conformers for (8*R*,9*R*,10*R*,11*S*)-**4**. 45
Figure S27. B3LYP/6-31G(d) optimized lowest energy conformers for (6*S*,7*S*,8*S*)-**2**. 46
Figure S28. HPLC analysis of *A. terreus* and identification of lovastatin. 47
Supplementary References 48

Methods

Bacterial strains, culture conditions, plasmids and reagents

All strains and primers used in this study are shown in Table S1 and S2, respectively, and details for plasmid construction are provided as following. DH5α chemically competent strains were cultured in LB with the appropriate antibiotics at 200 rpm and 37 °C. *C. arbuscula* was routinely cultured on PDA (potato dextrose agar) at 25°C for sporulation. For liquid fermentation, mycelia were inoculated into PDB (potato dextrose broth) and incubated at 25°C with shaking at 200 rpm.

Strain development

The procedure to develop *C. arbuscula* based on ATMT was performed according to the method developed previously (Cao et al., 2018). The *C. arbuscula ΔaurA hy-calR* mutant was originated from *C. arbuscula ΔaurA* transformed with the pFGL-gpdAp-calRA integration plasmid. The *C. arbuscula ΔaurAΔcalG* mutant was originated from *C. arbuscula ΔaurA hy-calR* transformed with the pFGL-hphR-calG-KO plasmid (Mao et al., 2015). *The C. arbuscula ΔaurAΔcalI* mutant was originated from *C. arbuscula ΔaurA hy-calR* transformed with the pFGL-hphR-calI-KO plasmid. The successful construction of all the aforementioned mutant strains was verified by confirmative PCR.

The *A. terreus ΔpyrG* mutant was constructed using a CRISPR/Cas9-mediated genome editing strategy. The *ΔpyrG* mutant was first generated using a two-plasmid system consisting of a Cas9 expression plasmid and a pUC18-based donor plasmid containing *pyrG* homologous arms. Transformants were screened on medium supplemented with uracil/uridine and 5-fluoroorotic acid (5-FOA, 2 mg/mL, Solarbio Science & Technology Co., Ltd., Beijing, China), and the deletion of *pyrG* was confirmed by confirmative PCR.

Subsequently, the *ΔpyrG* strain was transformed with an AMA1-based CRISPR/Cas9 plasmid carrying *lovB*-specific sgRNA and the *Aspergillus nidulans pyrG* gene as a transient selection marker, together with the *mRFP* gene flanked by the homologous arms of *lovB*. To track gene expression, the entire *lovB* ORF was replaced by the *mRFP* gene without leaving any extra genomic markers. In a parallel approach for the heterologous biosynthesis of **1**, the *lovB* ORF was similarly substituted with the *calI* gene. The generated *ΔpyrGΔlovB* mutant strains were then verified by PCR and sequencing, and the transient AMA1-based plasmid was subsequently eliminated through 5-

FOA counterselection.

Plasmid construction

The general procedures of molecular cloning were described as follows. The regular and mutated DNA fragments were amplified with Phanta Max Super-Fidelity DNA Polymerase (Vazyme Biotech Co., Ltd.) and primers (Tsingke Biotechnology Co., Ltd., Beijing, China), and assembled into fast-digested linearized plasmids by ClonExpress Ultra One Step Cloning Kit (Vazyme Biotech Co., Ltd., Nanjing, China) and Seamless Assembly Mix RK21020 (ABclonal Biotech Co., Ltd, Wuhan, China). Then, the plasmids were propagated in the competent *E. coli* DH5α (Tsingke Biotechnology Co., Ltd., Beijing, China) and further verified by Sanger sequencing. Plasmids and primers in this work were listed in Table S1 and Table S2.

For the *calRA* integration plasmid (pFGL-gpdAp-calRA), the *gpdAp* promoter and *calRA* gene were amplified using primers 1-4 and co-assembled into *Bam*HI-digested pFGL-hphR (BamHI and all other restriction enzymes used in this study were from Thermo Fisher Scientific, Waltham, MA, USA). For the *calG* and *calI* knockout plasmids (pFGL-hphR-calG-KO and pFGL-hphR-calI-KO), the ~1.5 kb left and right homologous arms of each gene were amplified using corresponding primers (5-8 for *calG*; 9-12 for *calI*). These arms were subsequently co-assembled into *Xba*I/*Hin*dIII-digested pFGL-hphR.

To construct plasmids for heterologous expression in *Aspergillus* strains, the AMA1-based replicative vector pFC902 (*pyrG* marker) was used. The primers 13 + 14 and 15 + 16 were used to amplify *afoGp* and *calI*, respectively, and assembled into *Pac*I-digested pFC902 to obtain pFC902-afoGp-calI. To construct the plasmids for CRISPR/Cas9-mediated genome editing in *A. terreus*, the *pyrG* donor plasmid pUC18-pyrG was first generated. The ~1.0 kb upstream and downstream homologous arms of *A. terreus pyrG* were amplified using primers 17-20, and then co-assembled into HindIII/*Bam*HI-digested pUC18 vector. For the Cas9/sgRNA expression plasmid targeting the *pyrG* locus, the *pyrG*-specific single guide RNA (sgRNA) spacer was synthesized and inserted into the *Nhe*I-digested pFC332N (NeoR marker) via standard ligation, yielding pFC332N NeoR-pyrG-sp. To achieve the markerless replacement of *lovB* with *calI* in *A. terreus*, the AMA1-based donor plasmid pFC902N-lovAB L+R calI was constructed. The *lovB*-specific sgRNA spacer, homologous arms of *lovB* and the *calI* expression cassette were amplified using primers 21-26. These three fragments were co-assembled into the *Mss*I-digested sgRNA-containing vector to yield the final

pFC902N-lovAB L+R calI plasmid

For construction of expression plasmids under the control of different promoters in *A. oryzae*, the primers 27 + 28 and 29 + 30 were used to amplify the *amy1p* and *calI* genes, and then co-assembled into *Pac*I-digested pFC902 to obtain pFC902-amy1p-calI. Similarly, the primers 31-33 were used to amplify the *amy2p* and *calI* genes, respectively, and assembled into *Pac*I-digested pFC902 to obtain pFC902-amy2p-calI. The primers 34-36 were used to amplify the *hlyAp* gene and assembled into *Bam*HI-digested pFC902 to obtain pFC902-hlyAp-calI. To construct the plasmid for the synthetic transcriptional cascade in *A. oryzae*, primers 37 + 38, 39 + 40, and 41 + 42 were used to amplify the *LovBp-calI*, *gpdAp-lovE*, and *tef1p-npgA* expression cassettes, respectively. These three fragments were then co-assembled into *Pac*I-digested pFC902 to obtain pFC902-LovBp-calI + gpdAp-lovE + tef1p-npgA. Similarly, the primers 43–46 were used to amplify the *afoGp-calI* and *gpdAp-afoA* genes, and assembled into *Pac*I-digested pFC902 to obtain pFC902-afoGp-calI + gpdAp-afoA + tef1p-npgA.

Compound purification

C. arbuscula ΔaurA + *hy-calR* was cultivated on a 16 L scale. The fermentation broth was filtered to remove the mycelia, and the supernatant was extracted with an equal volume of EtOAc for three times. All organic solvents and reagents (EtOAc, petroleum ether, acetone, CH_2Cl_2, MeOH, CH_3CN, and formic acid) were purchased from Sinopharm Chemical Reagent Co., Ltd. (Shanghai, China). The resulting EtOAc extract (9.8 g, dark brown) was subjected to a silica gel column with successive gradient elution of petroleum ether−acetone (9:1 to 1:1, v/v) and CH_2Cl_2-MeOH (9:1 to 0:10, v/v). The target fractions were subsequently processed by a Sephadex LH-20 column eluting with CH_2Cl_2-MeOH (1:1, v/v), and ultimately purified by preparative HPLC preparative HPLC eluting with a stepwise gradient CH_3CN-H_2O (0.1% formic acid) (20−70%, v/v, 60 min) to yield four high-purity compounds: **1** (20.8 mg, t_R = 50.2 min), **2** (5.2 mg, t_R = 31.6 min), **3** (4.5 mg, t_R = 28.5 min), and **4** (2.6 mg, t_R = 11.3 min). Compound **1** was identified as the known calcarisarbulide A, whereas compounds **2-4** were previously undiscovered in *C. arbuscula* and were named calcarisarbulides B−D, respectively. It should be noted that the 1.25 mg/L yield reported here corresponds to the amount of compound **1** purified after multi-step chromatography for structural characterization from the initial *C. arbuscula ΔaurA* + *hy-calR* strain, and does not represent the production titer of this strain. The yields of the engineered high-yield strains are presented in the

main text (Fig. 1D and Fig. 2C).

Structural determination of compounds 2−4

Compound **2** (calcarisarbulide B) was isolated as a white amorphous powder. Its molecular formula was determined to be $C_{13}H_{15}NO_4$ based on the HR-ESI-MS ion peak at *m/z* 250.1150 $[M + H]^+$ (Figure S2A), corresponding to seven degrees of unsaturation. The UV spectrum (Figure S2B) displayed a characteristic absorption peak at 233 nm, suggesting the presence of a conjugated system. Comprehensive analysis of the 1D and 2D NMR data (DEPT-135, COSY, HSQC, and HMBC, Figure S3-S8) revealed that **2** shares an identical planar structure with a previously reported analog (Table S4) (Abraham et al., 1995). However, distinct differences were observed in its stereochemistry. The large coupling constants ($J_{2,3}$ = 15.30 Hz and $J_{4,5}$ = 15.12 Hz) confirmed the trans-configurations of the $\Delta^{2,3}$ and $\Delta^{4,5}$ double bonds. According to the Karplus equation, the coupling constant between H-7 and H-8 ($J_{7,8}$ = 4.0 Hz) corresponds to a dihedral angle of approximately 44°, indicating a co-facial relationship. Furthermore, a significant NOE cross-peak (Figure S9) between H-6 and H-8 in the NOESY spectrum established their co-facial orientation. Consequently, the relative configuration of **2** was assigned as 6*S**, 7*S**, 8*S**.

Compound **3** (calcarisarbulide C) was obtained as colorless crystals. Its HRESIMS spectrum exhibited an $[M + H]^+$ peak at *m/z* 266.1104, establishing the molecular formula $C_{13}H_{15}NO_5$, which contains one more oxygen atom than **2** (Figure S10A). The UV absorption (λ_{max} 233 nm, Figure S10B) and NMR profiles were highly analogous to those of **2**, indicating a conserved core scaffold (Table S5). The only structural difference was the presence of an additional oxygen atom attached to the nitrogen of the five-membered heterocycle in **3**.

Compound **4** (calcarisarbulide D) was isolated as a colorless oil. The molecular formula was assigned as $C_{15}H_{21}NO_6$ based on the negative-ion HR-ESI-MS peak at *m/z* 310.1079 $[M-H]^-$, corresponding to six degrees of unsaturation (Figure S17A). Its UV spectrum exhibited a strong absorption at 300 nm, indicative of an extended conjugated system or an aromatic chromophore (Figure S17B). The 1D and 2D NMR data matched the planar structure of the known compound spirostaphylotrichin X (Figure S18-S23, Table S6, Wang et al., 2018). However, **4** displayed a completely opposite electronic circular dichroism (ECD) spectrum compared to spirostaphylotrichin X (Figure S24). For such molecules, the ECD signal is typically dominated by axial chirality. Given that the reported spirostaphylotrichin X possesses a 9*S* configuration, the

inverted ECD spectrum assigned the 9*R* configuration to **4**. The relative configuration was further elucidated via NOESY correlations (Figure S23): cross-peaks between 8-OH and 10-OH, as well as between 8-OH/H-10 and H-12, established the relative configuration of **4** as 8*R**, 9*R*, 10*R**, 11*S**. Hence, there are two possible candidate structures (8*R*,9*R*,10*R*,11*S*)-**4** and (8*S*,9*S*,10*S*,11*R*)-**4** for **4**, (6*S*,7*S*,8*S*)-**2** and (6*R*,7*R*,8*R*)-**2** for **2**, (8*R*,9*R*,10*R*,11*S*)-**4** and (6*S*,7*S*,8*S*)-**2** were randomly selected for theoretically study of their ECD. These conformers were reoptimized using DFT at the B3LYP/6-31G(d) level in gas phase using the Gaussian 09 program, and six conformers for (8*R*,9*R*,10*R*,11*S*)-**4** and two conformers for (6*S*,7*S*,8*S*)-**2** with relative Gibbs free energies in the range of 0-2 Kcal/mol were chosen for next calculation (Table S5). The B3LYP/6-31G(d) harmonic vibrational frequencies were further calculated to confirm their stability. For all the conformers, none of them showed imaginary frequencies after checking the resulted log files. The energies, oscillator strengths, and rotational strengths of the first 60 electronic excitations were calculated using the TDDFT methodology at the M062X/TZVP level in methanol (Table S8-S9). The ECD spectra were simulated by the overlapping Gaussian function (σ = 0.37 eV, σ = 0.4 eV for (8*R*,9*R*,10*R*,11*S*)-**4** and (6*S*,7*S*,8*S*)-**2**, respectively), in which velocity rotatory strengths of the first seven exited states for (8*R*,9*R*,10*R*,11*S*)-**4** and (6*S*,7*S*,8*S*)-**2** were adopted (Stephens and Harada, 2010). To get the final ECD spectra, the simulated spectra of the lowest energy conformers were averaged according to the Boltzmann distribution theory and their relative Gibbs free energy (ΔG) (Figure S25-S26).

In the 200-450 nm region (Figure S27), both the experimental ECD curve of **4** and the theoretical one for (8*R*,9*R*,10*R*,11*S*) configuration showed negative first and positive second Cotton effects. Therefore, qualitative analysis of the calculated and experimental ECD spectra allowed the assignments of the absolute configuration of **4** as (8*R*,9*R*,10*R*,11*S*).

As for **2** (Figure S28), the calculate ECD curve of (6*R*,7*R*,8*R*)-**2** fitted well with the experimental one, so the absolute configuration of **2** was assigned as (6*R*,7*R*,8*R*).

Table S1. All strains used in this study.

Strain	Genotype	Reference
Calcarisporium arbuscula	wild type, NRRL 3705, ATCC 46034	(Cao et al., 2023)
C. arbuscula ΔaurA	*aurB::hph*^{R}, the entire *aurB* was replaced with *hph*^{R}	(Mao et al., 2015)
C. arbuscula ΔaurA + hy-calR	*aurA::neo*^{R}, ectopic overexpression of *hy-calR*	This study
C. arbuscula ΔaurA + hy-calR ΔcalG	*calG::hyh*^{R}, the entire *calG* was replaced with *hyh*^{R}	This study
C. arbuscula ΔaurA + hy-calR ΔcalI	*calI::hyh*^{R}, the entire *calI* was replaced with *hyh*^{R}	This study
Aspergillus nidulans LO8030	*pyroA4, riboB2, ΔpyrG89, nkuA::argB;* multiple secondary metabolite gene clusters deleted	(Chiang et al., 2021)
A. nidulans+gpdAp-afoA+afoGp-calI-npgA	*A. nidulans* harboring the *afo*-based synthetic transcriptional cascade	This study
A. terreus	wild type	This study
A. terreusΔpyrG	Uracil/uridine auxotrophic strain	This study
A. terreusΔpyrGΔlovB + calI	*lovB::calI, the entire lovB was replaced with calI*	This study
A. terreusΔpyrGΔlovB + gpdAp-lovE + lovBp-calI+ tef1p-npgA	*A. terreus ΔlovB* harboring the *lov*-based synthetic transcriptional cascade	This study
Aspergillus oryzae RIB40	wild type, ATCC 42149	(Nguyen et al., 2016)
A. oryzae ΔpyrG	wild-type, *A. oryzae* with *pyrG* gene deletion	(Nguyen et al., 2016)
A. oryzae ΔpyrG + amy1p-calI	*A. oryzae ΔpyrG* harboring plasmid *amy1p-calI*	This study
A. oryzae ΔpyrG +amy2p-calI	*A. oryzae ΔpyrG* harboring plasmid *amy2p-calI*	This study
A. oryzae ΔpyrG+hlyAp-calI	*A. oryzae ΔpyrG* harboring *plasmid hlyAp-calI*	This study
A. oryzae ΔpyrG + gpdAp-lovE + lovBp-calI+ tef1p-npgA	*A. oryzae ΔpyrG* harboring the *lov*-based synthetic transcriptional cascade	This study
A. oryzae ΔpyrG + gpdAp-afoA + afoGp-calI + tef1p-npgA	*A. oryzae ΔpyrG* harboring the *afo*-based synthetic transcriptional cascade	This study

Table S2. Plasmids used in this study.

Name	Description	Reference
pFGL-hphR	*E. coli-A. tumefaciens* shuttle vector, hygromycin marker	(Cao et al., 2018)
pFGL-gpdAp-calRA	. *gpdAp* driving expression of hybrid transcription factor *calRA*; integration at *aurA* locus	This study
pFGL-hphR-calG-KO	*E. coli-A. tumefaciens* shuttle vector, hygromycin resistance marker *calG* left arm (1.5 kb) and right arm (1.5 kb) cloned into *Xba*I/*Hin*dIII-digested pFGL-hphR	This study
pFGL-hphR-calI-KO	*E. coli-A. tumefaciens* shuttle vector, hygromycin resistance marker *calI* left arm (1.5 kb) and right arm (1.5 kb) cloned into *Xba*I/*Hin*dIII-digested pFGL-hphR	This study
pFC902-afoGp-calI	*E. coli-A. nidulans* shuttle vector, *gpdAp* driving *afoA* expression	This study
pUC18-pyrG	*E. coli-A. terreus* shuttle vector, the donor plasmid constructed based on the pUC18 backbone, containing the homologous arms of *A. terreus pyrG*	This study
pFC332N NeoR-pyrG-sp	*E. coli-A. terreus* shuttle vector, the Cas9/sgRNA expression plasmid targeting the *A. terreus pyrG* locus, conferring neomycin resistance (*neo*^{R})	This study
pFC902N-lovAB L+R calI	*E. coli–A. terreus* shuttle vector harboring a *lovB*-specific sgRNA, the *AnpyrG* marker, and *lovB* L-arm + *calI* + *lovB* R-arm for targeted gene replacement in *A. terreus*.	This study
pFC902-amy1p-calI	*E. coli-A. oryzae* shuttle vector, *pyrG* marker; *amy1p-calI* gene cloned into *Bam*HI-digested-pFC902	This study
pFC902-amy2p-calI	*E. coli-A. oryzae* shuttle vector, *pyrG* marker; *amy2p-calI* gene cloned into *Bam*HI-digested-pFC902	This study
pFC902-hlyAp-calI	*E. coli-A. oryzae* shuttle vector, *pyrG* marker; *hlyAp-calI* gene cloned into *Bam*HI-digested-pFC902	This study
pFC902-LovBp-calI + gpdAp-LovE + tef1p-npgA	*E. coli-A. oryzae* shuttle vector, AMA1-based, *pyrG* marker, *afoGp-calI-npgA*, *gpdAp-afoA*, and *tef1p-npgA* expression plasmid	This study
pFC902-afoGp-calI + gpdAp-afoA + tef1p-npgA	*E. coli-A. oryzae* shuttle vector, AMA1-based, *pyrG* marker, *afoGp-calI-npgA*, *gpdAp-afoA*, and *tef1p-npgA* expression plasmid	This study

Table S3. All primers used in this study.

No.	Sequence
1	CAACACTGCGATTGAGTCTG
2	GCTCCATTTTGTCCATCCGA
3	TGCTCCGTAACACCCAATAC
4	TGATCGCCTGTGTGACAAAG
5	CAGGGTATACTTTGAGGGTG
6	CAGGAGATGTTGCTGAAGTC
7	TGCTCCGTAACACCCAATAC
8	CACTGGCGGCTATGTTCAAG
9	CGTAGTGTAGACCTGCGAAG
10	AGTGTGGGATGTCACCTCTG
11	CTCGTCGAATCGTCGATAG
12	AAATGCCTTCTGTGGGG
13	TGTCTAGTATAACGCCATATC
14	AAATGCCTTCTGTGGGG
15	CTAAGGTTCTAGGTACTTAG
16	CTTGCTTCGACTTCGTTTG
17	ACGACGGCCAGTGCCAAGCTTATTGGACGATGACTTTGCCG
18	TTACCCCTCCATCGCCGACCGTTAGTATAGTATAGACGAT
19	GGTCGGCGATGGAGGGGTAA
20	AGCTCGGTACCCGGGGATCCAGGCGCAGGGCAAGGGCCGA
21	GCTCATACGATACGACTGGAGTTTTAGAGCTAGAAATAGCAA
22	TATTTCTAGCTCTAAAACAAGCGCATGGGGACAGCTAATGCATCATCCGTGAATC
23	TATACTGGGCCCGGGAAGATTGTGGTATATCCAGG
24	ACCTGGTCTCCTAGCCTTAATTAACAGTAGTCTACCCCAGAAG
25	GGCTAGGAGACCAGGTG
26	TTCTATGTTTGCGCCATCATCTTCGCGATCTAAGC
27	TGAGCACTATCCAGACGAG
28	AGACGGAGAAACCAGAACCG
29	CCTGGTGGAGGATCCGAC
30	TCATCTTCGCGATCTAAGCC
31	ATGTCTCATTCTCCAACCG
32	TCAAAACGAAATCTCCTCCC
33	ATGGCGTGTCCCACCAGACG
34	CGTGGTTGATGGCGACTTG
35	AATGACTGGTCCGTCCG
36	AATGACTGGTCCGTCCG
37	AACACTACGTGCGAGTGTTTAAACGGCTAGGAGACCAGGTGTG
38	GCTGTTTGCAGTAGGAC
39	TCCTACTGCAAACAGCATGACGAAAACGACATCGTC
40	GGTCATGTAATATCACACGCGTTAATTAATATCTCTGTCGCCGTCA
41	CCGGGAAGATCTGTTTGCATATGTACGGAGTATCTG
42	ACTCGCACGTAGTGTTTCGAGTACCTACTTGAG

43	TACTGGGCCCGGGAAGATCTAATGACTGGTCCGTCCG
44	GGTGCCTTGTGGATGG
45	CATCCACAAGGCACCATGACGAAAACGACATCGTC
46	ACATGACCGATGCAGCTCATCTTCGCGATCTAAGCC

Table S4. The chemical shift of compound 2 in CD_3OD.

Position	δ_H	δ_C
1	1.76 (3H, d, 6.6)	16.87, CH_3
2	5.80 (1H, m)	130.43, CH
3	6.34 (1H, dd, 15.30, 10.68)	134.61, CH
4	6.07 (1H, dd, 15.12, 11.04)	131.10, CH
5	5.80 (1H, m)	124.09, CH
6	4.82 (1H, m)	83.22, CH
7	3.82 (1H, t, 4.38)	71.72, CH
8	4.37 (1H, d, 3.84)	63.05, CH
9		105.39, C
10		138.11, C
11		159.52, C
12	5.03 (2H, s)	92.61, CH_2
13		171.25, C

Table S5. The chemical shift of compound 3 in CD_3OD.

Position	δ_H	δ_C
1	1.75 (3H, d, 6.6)	16.85, CH_3
2	5.80 (1H, m)	130.42, CH
3	6.33 (1H, dd, 15.08, 10.60)	134.77, CH
4	6.08 (1H, dd, 15.12, 11.04)	131.13, CH
5	5.80 (1H, m)	123.96, CH
6	4.78 (1H, m)	83.28, CH
7	3.82 (1H, t, 4.40)	71.91, CH
8	4.32 (1H, d, 4.40)	62.71, CH
9		103.09, C
10		139.19, C
11		156.16, C
12	5.07 (2H, s)	91.50, CH_2
13		167.41, C

Table S6. The chemical shift of compound 4 in $CDCl_3$.

Position	δ_H	δ_C
1	1.06 (3H, t, 7.50)	13.70, CH_3
2	2.28, 2.39 (2H, m)	21.90, CH_2
3	5.86 (1H, t, 7.08)	144.88, CH
4		128.48, C
5	7.46 (1H, d, 10.08)	142.67, CH
6	6.05 (1H, d, 10.14)	122.94, CH
7		197.64, C
8	4.80 (1H, s)	72.85, CH
9		59.89, C
10	4.20 (1H, d, 11.70)	70.27, CH
11		89.32, C
12	1.65(3H, s)	17.56, CH_3
13		167.83, C
14	3.47(3H, s)	52.17, CH_3
15	3.95(3H, s)	64.65, CH_3
8-OH	3.78 (1H, brs)	
10-OH	2.55 (1H, d, 11.88)	

Table S7. Energy analysis for (8*R*,9*R*,10*R*,11*S*)-4 and (6*S*,7*S*,8*S*)-2.

Conf.	G (Hartree)	ΔG (Kcal/mol)	Boltzmann Distribution
	(8*R*,9*R*,10*R*,11*S*)-**4**		
**C1**	-1089.928276	0	0.443433539
**C2**	-1089.925272	1.88504004	0.018381441
**C3**	-1089.928208	0.04267068	0.412605253
**C4**	-1089.926319	1.22803707	0.055745756
**C5**	-1089.925379	1.81789647	0.020588336
**C6**	-1089.926202	1.30145574	0.049245675
(6*S*,7*S*,8*S*)-**2**			
**C1**	-859.779351	0	0.931902677
**C2**	-859.776882	1.54932219	0.068097323

Table S8. Calculated ECD Data for (8*R*,9*R*,10*R*,11*S*)-4.

States	C1		C2		C3	
	Excitation energies(eV)	Rotatory Strengths*	Excitation energies(eV)	Rotatory Strengths*	Excitation energies(eV)	Rotatory Strengths*
1	3.9652	-33.6414	3.9618	-35.4094	3.9629	-37.3023
2	4.2873	86.6486	4.2688	74.1027	4.2829	99.9937
3	5.3511	2.5415	5.3225	-3.0203	5.348	3.4124
4	5.7109	3.4007	5.5962	12.7998	5.7069	3.6487
5	5.8516	-55.2568	5.7558	9.9611	5.8493	-55.6744
6	5.9351	44.9209	6.0182	-27.1329	5.9265	43.7243
7	6.2777	-13.246	6.3041	27.4343	6.2756	-13.7702
8	6.3872	37.0643	6.3539	6.734	6.4097	8.1102
9	6.4985	7.9596	6.4367	2.6171	6.4826	0.978
10	6.5998	0.6708	6.5703	-42.4886	6.5961	-18.405
11	6.6292	-40.5758	6.6209	-24.3322	6.6177	-32.3833
12	6.7274	-29.0752	6.8834	75.064	6.7429	-31.2076
13	6.8324	32.8524	6.9919	-21.5177	6.8369	30.5596
14	7.1958	-21.3973	7.1686	20.609	7.1458	13.7388
15	7.2182	18.3666	7.2162	-31.1248	7.1984	-23.1041
16	7.3545	11.545	7.3414	13.7494	7.3667	3.5248
17	7.43	-24.3815	7.3819	14.4106	7.4311	-25.6937
18	7.5324	1.4853	7.4899	-6.4333	7.5303	1.309
19	7.6204	-49.7847	7.5286	-57.6446	7.6716	-22.4757
20	7.6738	-1.5183	7.6453	-2.8443	7.7025	-19.0109
21	7.7121	1.7763	7.6563	-8.0865	7.7151	1.1333
22	7.7879	2.1214	7.7437	35.1174	7.7852	9.2688
23	7.8825	24.7249	7.8065	-6.7508	7.8542	-16.2687
24	7.9129	8.223	7.8487	23.1747	7.9064	25.4261
25	7.9926	7.2461	7.935	6.0889	7.9291	-2.0548
26	8.0127	-20.997	7.9852	-11.1043	8.0137	-20.0958
27	8.0292	1.3202	8.0432	-0.7247	8.0231	3.1068
28	8.0602	20.3728	8.1184	-0.9387	8.0439	3.1988
29	8.1324	-5.5393	8.1204	-5.0458	8.117	-15.4041
30	8.1547	-22.524	8.1975	39.5704	8.1492	-17.347
31	8.2245	3.8167	8.2323	-19.0175	8.2189	-6.8945
32	8.2882	-25.2857	8.2908	-42.8303	8.2711	29.0572
33	8.3533	13.9172	8.3182	-7.9373	8.3468	11.7105
34	8.4083	10.7009	8.346	7.1361	8.4242	-19.3667
35	8.4236	-26.698	8.4151	-7.6174	8.4308	-16.0366
36	8.5064	-30.8122	8.4242	-4.4692	8.4686	-9.2163
37	8.5156	-2.0453	8.4714	-33.6079	8.501	2.3321
38	8.5475	-33.7194	8.5186	-9.3271	8.5444	-10.3483
39	8.5781	-26.1249	8.5457	50.4568	8.5924	-15.5112

40	8.626	62.6557	8.6404	-19.5657	8.6243	49.4409
41	8.6562	-40.7778	8.6768	9.6315	8.6703	13.2792
42	8.6877	9.7545	8.6928	6.9909	8.7036	-29.2559
43	8.7263	-46.489	8.7302	4.2571	8.7741	-6.8951
44	8.756	11.4364	8.7514	2.7962	8.7874	20.1117
45	8.7826	27.1877	8.7626	-18.4587	8.7973	-10.788
46	8.8302	17.3228	8.8189	-2.4481	8.8238	10.7234
47	8.845	-1.5219	8.8593	19.2778	8.8469	-53.1647
48	8.8612	-37.3599	8.9105	-11.9663	8.8756	13.5339
49	8.895	-5.5247	8.9286	6.6608	8.9016	7.349
50	8.9434	10.4958	8.9828	-4.6107	8.9721	19.7372
51	8.9677	8.4924	8.9867	1.3874	8.9935	5.5444
52	9.0012	9.7569	9.0594	10.1976	9.0205	6.1686
53	9.0412	-41.0286	9.0766	-11.6341	9.0573	6.6721
54	9.0861	2.0326	9.106	-27.2107	9.0884	18.418
55	9.0908	-5.4294	9.1132	-20.1405	9.1012	-37.2261
56	9.1318	2.425	9.1535	4.8569	9.1433	6.0278
57	9.1937	-22.4955	9.1876	14.2883	9.2007	-19.928
58	9.2216	-12.8041	9.215	5.894	9.219	13.0013
59	9.2335	24.3019	9.2526	26.4201	9.2453	34.9449
60	9.2777	-1.9182	9.2621	72.6737	9.2865	-50.3018

States	**C4**		**C5**		**C6**	
	Excitation energies(eV)	Rotatory Strengths*	Excitation energies(eV)	Rotatory Strengths*	Excitation energies(eV)	Rotatory Strengths*
1	3.9596	-33.0697	3.9595	-39.1496	3.9576	-36.2824
2	4.3016	49.3191	4.2641	87.769	4.2984	64.7483
3	5.5161	2.3296	5.3195	-1.9514	5.5107	3.914
4	5.6494	45.6461	5.5901	12.3993	5.6421	49.1429
5	5.8245	-28.3581	5.7545	10.4429	5.8237	-30.7325
6	6.0047	7.058	6.0096	-26.874	6.0008	6.1339
7	6.2708	-11.649	6.3085	-17.5815	6.2752	-12.7714
8	6.3647	45.4405	6.3569	28.8954	6.3866	-18.6846
9	6.4668	-9.2013	6.4376	4.548	6.4641	18.2425
10	6.5775	0.3534	6.5635	-64.6022	6.5691	0.0108
11	6.6696	-19.2377	6.6327	-34.7446	6.6748	-24.3896
12	6.9517	-60.2965	6.884	89.0298	6.9536	-114.2031
13	6.9766	17.1961	6.9734	-36.9076	6.9617	71.1639
14	7.1304	11.8926	7.1328	29.0686	7.1181	15.916
15	7.178	18.4381	7.2124	-37.2886	7.1648	1.9034
16	7.3438	8.303	7.3571	5.4071	7.3514	3.3066
17	7.419	-4.7429	7.3816	10.6879	7.4126	-1.8525
18	7.5379	-49.3745	7.4934	-9.4106	7.5824	-11.5409

19	7.5766	10.9868	7.5826	-35.2425	7.6155	-14.5348
20	7.6162	-15.5214	7.6507	-5.2324	7.6235	-5.9607
21	7.6633	39.7508	7.6577	-13.2997	7.6692	17.8938
22	7.8085	13.6241	7.7456	22.196	7.7877	5.6845
23	7.8569	-6.224	7.7924	27.5242	7.814	11.9974
24	7.8632	11.1701	7.8323	8.7049	7.8719	0.812
25	7.8884	12.4806	7.905	9.549	7.8819	1.6187
26	7.9497	-16.6062	7.9511	-37.0548	7.9474	-12.4896
27	7.968	-7.0173	8.0132	-9.9189	7.9685	-12.7223
28	8.0473	-4.1959	8.0942	-42.1203	8.0431	-27.5099
29	8.0615	39.5771	8.1159	-4.3115	8.0576	49.4998
30	8.17	-18.5294	8.1739	10.4381	8.1538	-19.0894
31	8.2387	21.9135	8.2153	11.1551	8.211	40.3271
32	8.284	18.6691	8.2905	-5.733	8.2719	36.3574
33	8.3372	15.5593	8.3435	-22.8257	8.3496	1.1078
34	8.4015	-35.4156	8.3498	6.2089	8.3964	-10.5183
35	8.4082	-38.3571	8.3795	-5.5117	8.4183	-11.4233
36	8.4655	68.9177	8.4228	0.8347	8.4645	-0.3952
37	8.4833	-39.6925	8.4695	0.8616	8.4808	-3.5366
38	8.512	-25.3211	8.5202	-18.032	8.5212	-26.7387
39	8.5878	61.4582	8.5738	21.1984	8.5903	54.8324
40	8.6562	34.2052	8.6775	66.8434	8.6501	26.518
41	8.7238	-1.922	8.6871	0.8116	8.7413	-7.6129
42	8.7462	4.4794	8.695	30.3218	8.7626	-0.0286
43	8.7781	-33.1049	8.7502	-0.8927	8.7722	-38.4603
44	8.792	-7.5437	8.7742	-8.3214	8.8129	9.8865
45	8.8307	-26.2262	8.783	-12.1779	8.8285	-13.1
46	8.8625	-4.3616	8.8188	-14.5101	8.8593	-3.0869
47	8.8864	-3.8802	8.8637	-6.8804	8.9004	16.3011
48	8.907	0.3975	8.9012	7.9887	8.9219	7.7262
49	8.9245	-1.4977	8.9426	21.7146	8.9433	-16.8932
50	8.9607	-15.9073	9.0029	-25.3167	8.9931	-2.6733
51	8.9969	16.4223	9.0132	35.3809	9.0066	11.0206
52	9.0455	-8.0224	9.047	11.773	9.0452	-15.0639
53	9.054	16.6608	9.078	-33.9141	9.0835	40.2461
54	9.0994	10.4285	9.1162	6.9119	9.1109	12.2708
55	9.1525	38.9322	9.1197	-39.0627	9.143	89.23
56	9.1659	43.4258	9.1578	14.9172	9.1783	27.3554
57	9.1848	-29.937	9.188	18.0965	9.1917	-9.4658
58	9.2262	-7.3238	9.2147	61.2843	9.224	-15.7501
59	9.2709	12.5823	9.2497	9.8999	9.2705	-11.1806
60	9.2937	13.536	9.2667	23.6229	9.285	58.5969

* R(velocity)   10**-40 erg-esu-cm

Table S9. Calculated ECD Data for (6*S*,7*S*,8*S*)-2.

States	C1		C2	
	Excitation energies(eV)	Rotatory Strengths*	Excitation energies(eV)	Rotatory Strengths*
1	4.4985	-4.038	4.5218	10.717
2	4.9428	-150.6399	4.9343	-85.5224
3	5.0864	143.5898	5.0308	14.3779
4	5.3352	-4.8878	5.0822	160.8814
5	5.507	15.2839	5.5882	-74.1499
6	6.3999	4.3606	6.4539	-9.0318
7	6.462	-9.1659	6.7504	-2.3217
8	6.8149	-4.2559	6.7929	-4.9992
9	6.8326	-0.1618	7.0977	0.0643
10	7.2487	2.4991	7.1755	-4.1779
11	7.3179	40.0343	7.2388	-2.3289
12	7.3194	-40.0897	7.3176	-2.8269
13	7.4197	-16.9026	7.4068	-4.8176
14	7.4615	-2.0905	7.4271	11.8447
15	7.4952	3.7656	7.4846	-8.5416
16	7.5336	34.5983	7.5112	-0.0779
17	7.6122	17.4388	7.5363	25.4773
18	7.6849	-13.4465	7.6203	27.7678
19	7.757	43.3056	7.712	4.1012
20	7.7887	14.0813	7.7326	5.7661
21	7.861	1.0084	7.8026	3.8756
22	7.8709	0.5796	7.8572	-6.8079
23	7.9388	10.3936	7.9563	-12.0769
24	7.9785	-34.7285	7.9664	-8.9797
25	8.0067	-24.1578	7.9933	-24.5051
26	8.1044	38.5271	8.0481	9.3849
27	8.1554	2.8109	8.0621	18.891
28	8.2181	6.4664	8.1736	20.6769
29	8.2689	7.2412	8.2455	4.5873
30	8.2948	-17.502	8.272	18.9376
31	8.3714	-0.5968	8.2784	-22.3588
32	8.4071	-38.9756	8.3214	-18.5016
33	8.4377	3.1073	8.397	-2.6472
34	8.4846	-4.5808	8.4373	31.9367
35	8.5144	-14.4559	8.4639	-31.7606
36	8.5406	13.4134	8.4701	10.6186
37	8.5633	2.9965	8.5301	-8.3138
38	8.5989	11.1199	8.5764	-11.5432
39	8.6238	4.7944	8.5884	44.6472

40	8.6736	-7.8643	8.6624	21.9519
41	8.7335	6.1334	8.6807	11.0855
42	8.7392	1.7634	8.749	-19.7158
43	8.8376	1.6943	8.7725	-30.1387
44	8.8716	35.1671	8.8193	-38.1638
45	8.9132	2.5919	8.8593	-10.9085
46	8.974	16.5925	8.8822	67.3411
47	8.9955	12.4157	8.9672	1.0195
48	9.0235	-4.5061	8.9835	19.9423
49	9.0635	-12.6568	9.0059	15.4939
50	9.0701	16.3149	9.0374	-37.3072
51	9.103	-1.4471	9.0716	-7.5198
52	9.1365	-18.1005	9.1127	-11.7495
53	9.1429	0.0509	9.1481	-1.922
54	9.2176	-9.6452	9.2323	4.9483
55	9.2373	8.6357	9.2499	-5.5664
56	9.3122	-24.6111	9.2722	-4.8772
57	9.3801	6.4217	9.3221	-4.2187
58	9.4093	6.1717	9.3537	8.7074
59	9.4214	-9.9931	9.3637	55.5453
60	9.4336	13.7259	9.4106	-63.4836

* R(velocity)   10**-40 erg-esu-cm

Table S10. BLAST analysis of *cal* GC homologs in *A. nidulans* LO8030 and *A. terreus* XM381.

Gene	*A. nidulans* LO8030	*A. terreus* XM381
calI (PKS-NRPS)	XP_659388.1 (non-reducing PKS pyr2) E=0.0, 36% identity, 52% positives	Acitbctg00000001G00022300.1 E=0.0, 34% identity, 52% positives
calF (HNO)	XP_660252.1 (putative isoamyl alcohol oxidase) E=2e-176, 47% identity, 63% positives	Acitbctg00000000G00012960.1 E=2e-177, 48% identity, 62% positives
calD (FAD)	XP_664685.1 (FAD-dependent oxidoreductase) E=4e-126, 39% identity, 57% positives	Acitbctg00000002G00033370.1 E=2e-127, 40% identity, 56% positives
calC (MFS)	XP_681728.1 (MDR family MFS transporter) E=2e-137, 48% identity, 66% positives	Acitbctg00000001G00029580.1 E=1e-122, 37% identity, 59% positives
calE (P450)	XP_681779.1 (uncharacterized protein) E=5e-43, 35% identity, 58% positives	Acitbctg00000001G00030120.1 E=5e-104, 35% identity, 55% positives
calK (AAR)	XP_657981.1 (aldo/keto reductase) E=2e-37, 29% identity, 49% positives	Acitbctg00000008G00100270.1 E=1e-76, 40% identity, 61% positives
calJ (HP)	XP_050468961.1 (amidase) E=8e-16, 25% identity, 42% positives	Acitbctg00000005G00069210.1 E=1e-48, 33% identity, 50% positives
calG (ACO)	XP_681162.2 (isopenicillin N synthase family dioxygenase) E=3e-65, 42% identity, 57% positives	Acitbctg00000000G00000490.1 E=2e-48, 36% identity, 53% positives
calH (FMO)	XP_664354.1 (FAD-dependent oxidoreductase) E=1e-25, 29% identity, 44% positives	Acitbctg00000002G00033330.1 E=1e-35, 27% identity, 43% positives
calN (HP)	XP_663611.1 (SKI complex RNA helicase) E=9e-20, 30% identity, 51% positives	Acitbctg00000005G00075270.1 E=8e-20, 30% identity, 51% positives
calM (HP)	XP_659946.1 (RTA1 domain-containing protein) E=0.076, 29% identity, 40% positives	Acitbctg00000003G00045210.1 E=1.2, 31% identity, 51% positives
calL (AH)	XP_664678.1 (SDR family oxidoreductase) E=0.005, 48% identity, 58% positives	Acitbctg00000000G00005180.1 E=0.005, 48% identity, 61% positives
calR (TF)	XP_662230.1 (Zn2Cys6 transcription factor) E=8e-7, 39% identity, 51% positives	No significant hit (E > 1e-5)
calB (OMT)	XP_660213.1 (uncharacterized protein) E=2e-25, 33% identity, 48% positives	No significant hit (E > 1e-5)

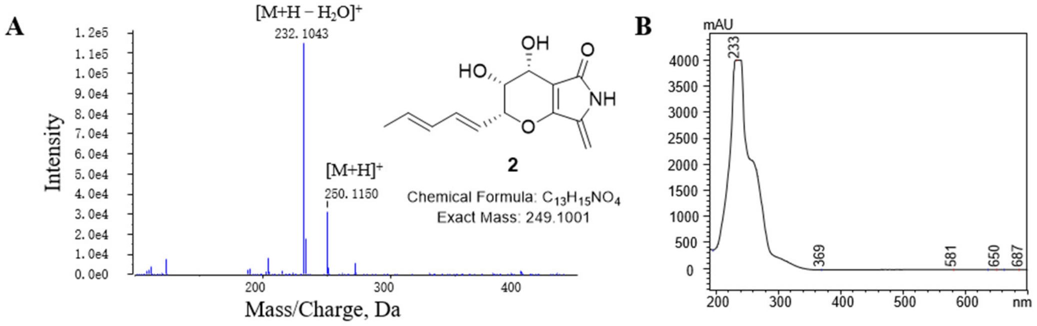

Figure S1. HRMS and UV spectra of compound 2.

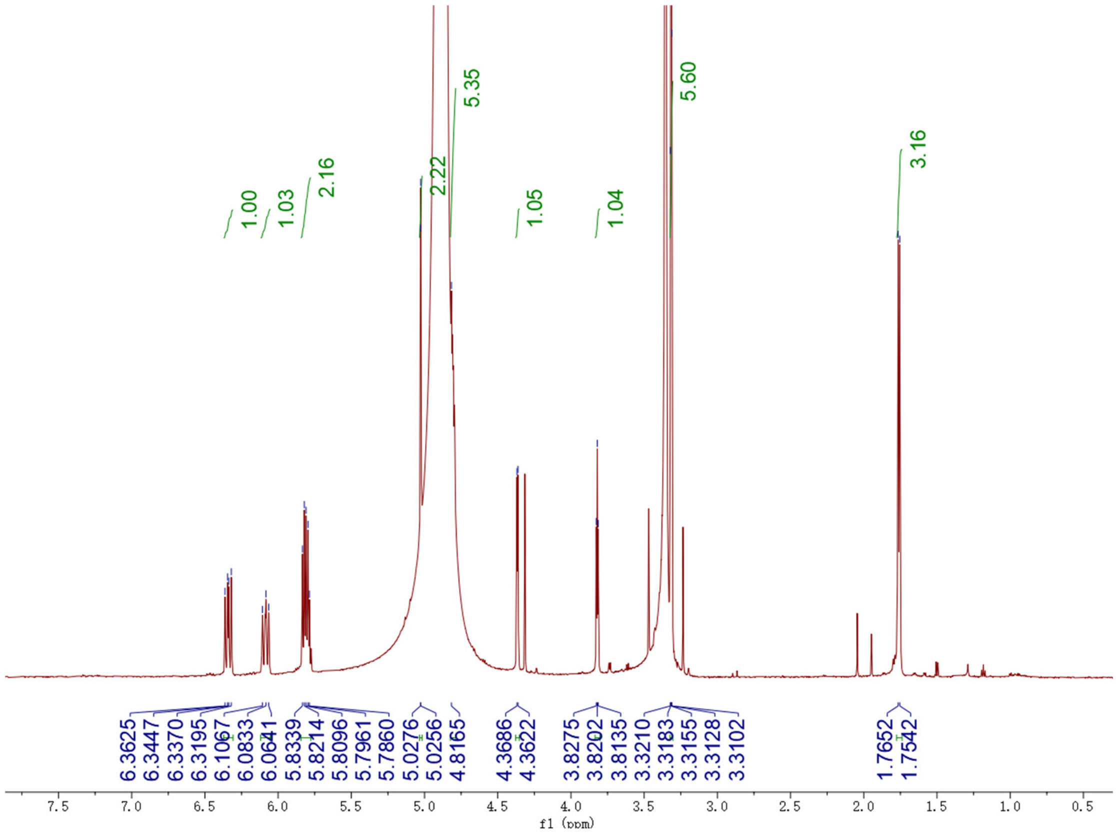

Figure S2. ^{1}H NMR of compound 2 in CD_3OD at 600 MHz.

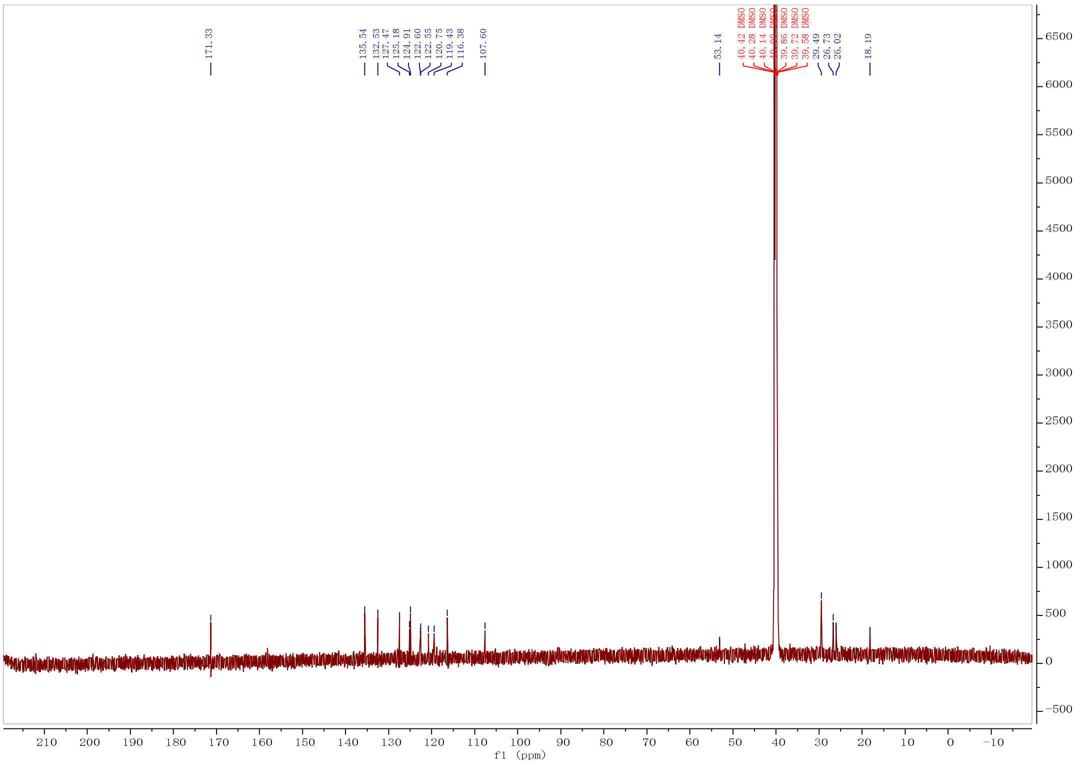

Figure S3. ^{13}**C NMR of compound 2 in CD$_3$OD at 150 MHz.**

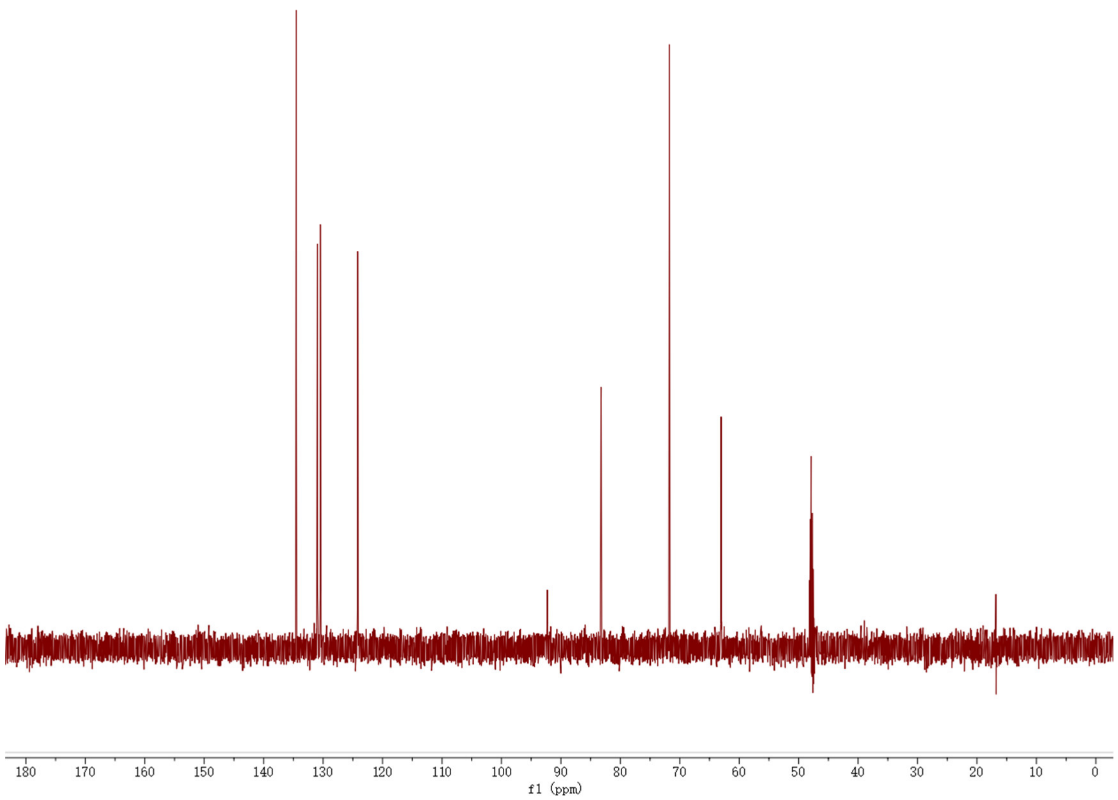

Figure S4. DEPT135 NMR of compound 2 in CD_3OD at 150 MHz.

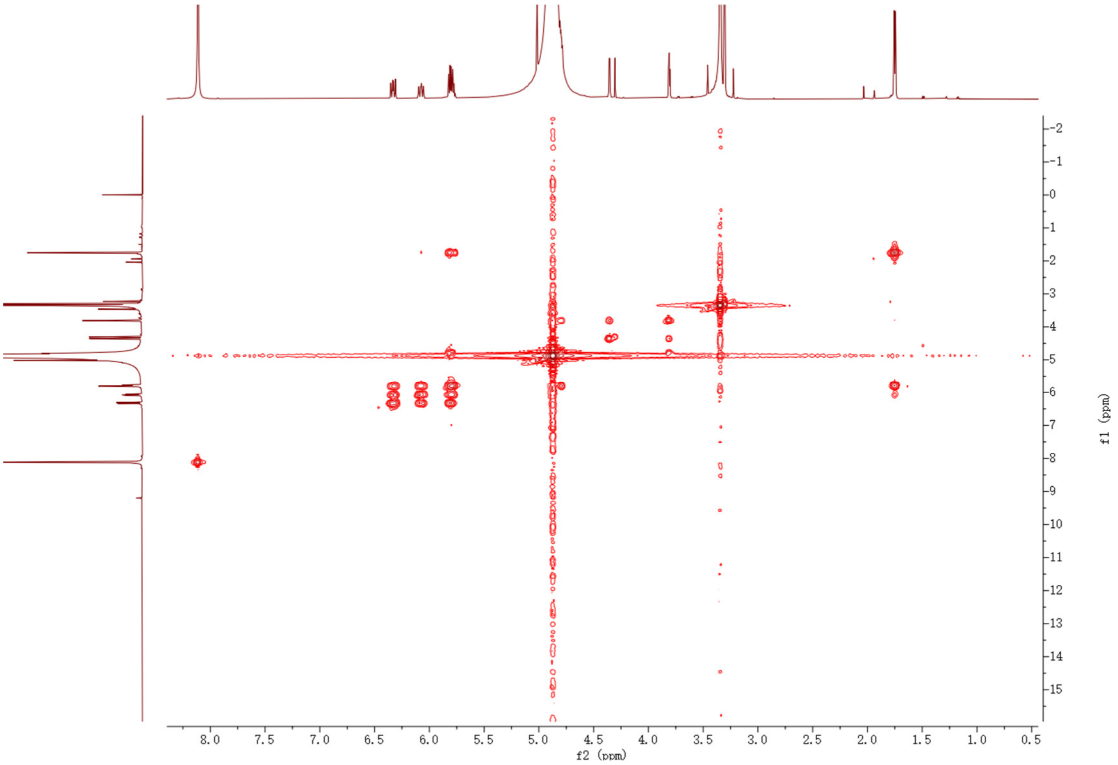

Figure S5. ^{1}H-^{1}H COSY of compound 2 in CD_3OD.

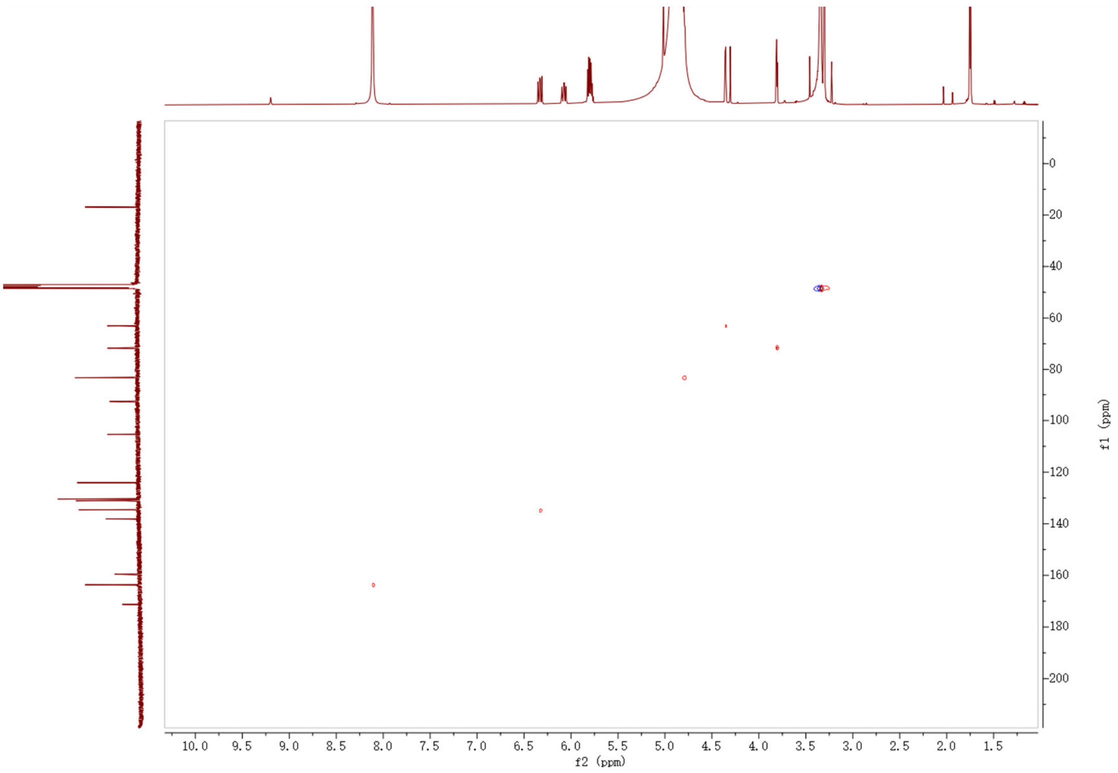

Figure S6. HSQC of compound 2 in CD_3OD.

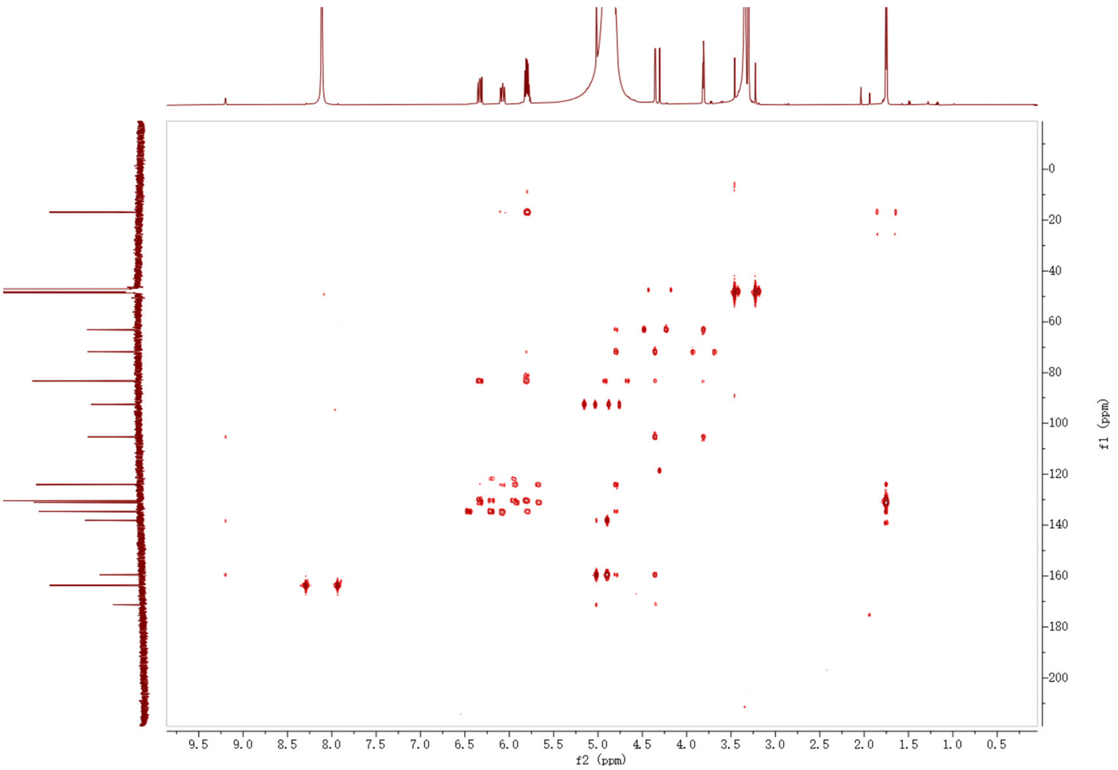

Figure S7. HMBC of compound 2 in CD_3OD.

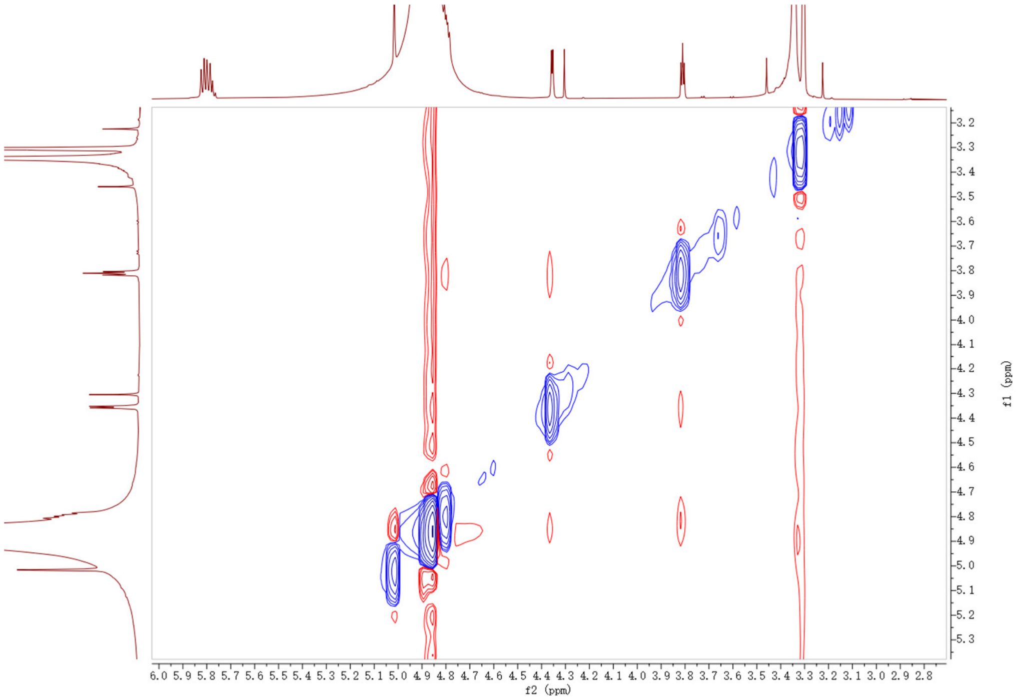

Figure S8. NOESY of compound 2 in CD_3OD.

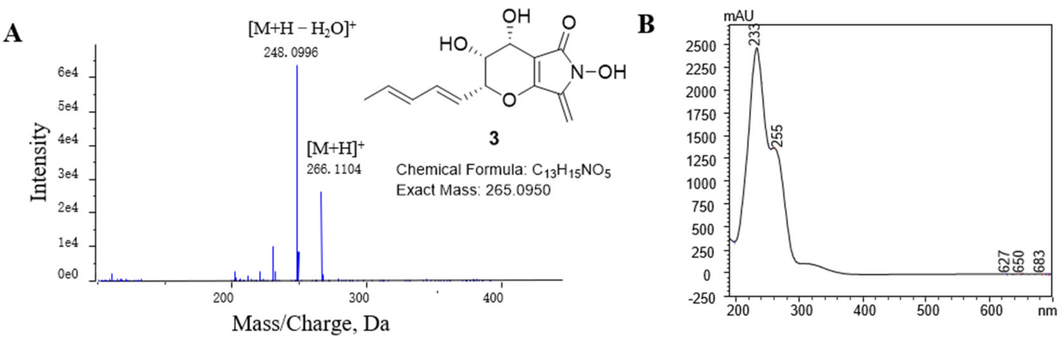

Figure S9. HRMS and UV spectra of compound 3.

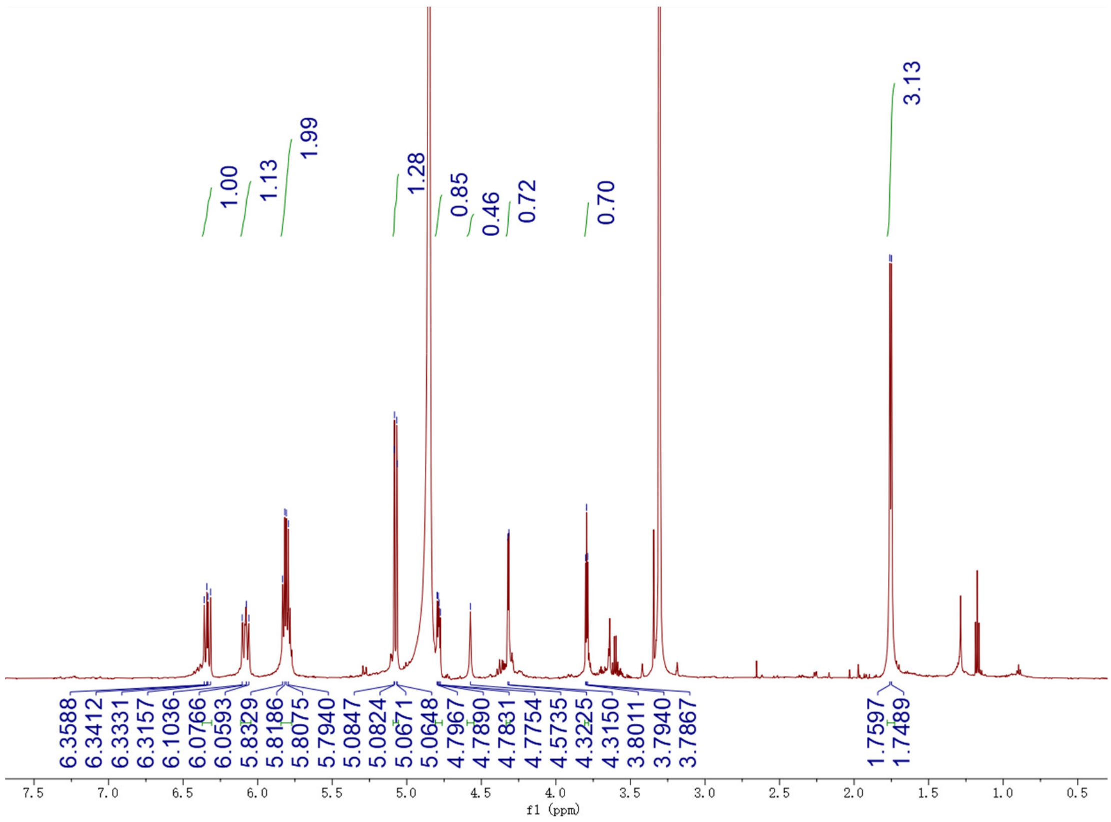

Figure S10. ^{1}H NMR of compound 3 in CD_3OD at 600 MHz.

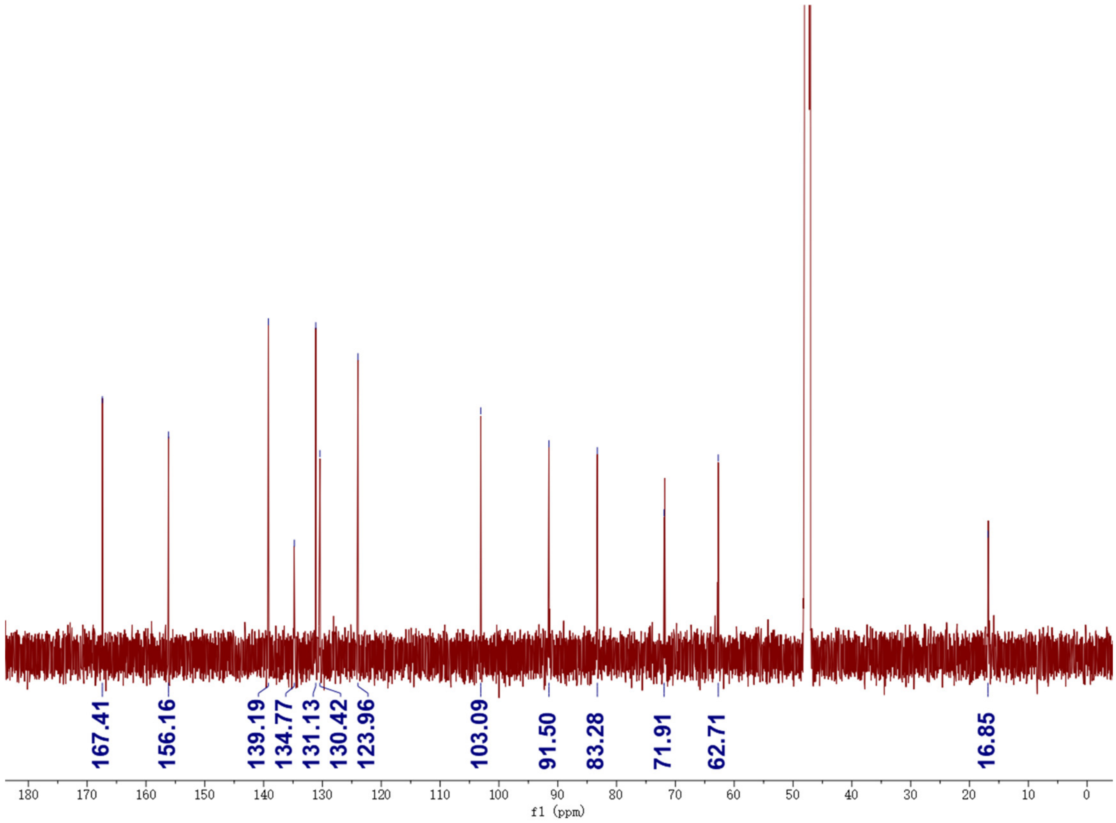

Figure S11. ^{13}C NMR of compound 3 in CD_3OD at 600 MHz.

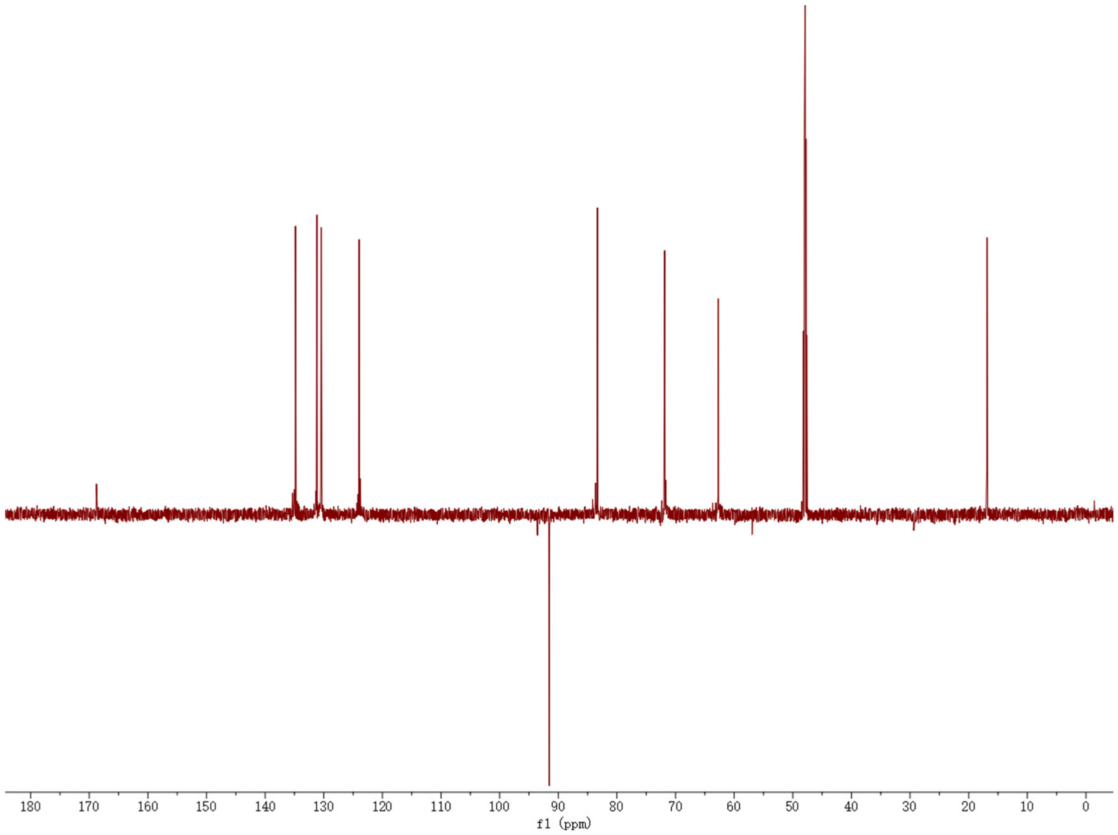

Figure S12. DEPT135 of compound 3 in CD_3OD at 150 MHz.

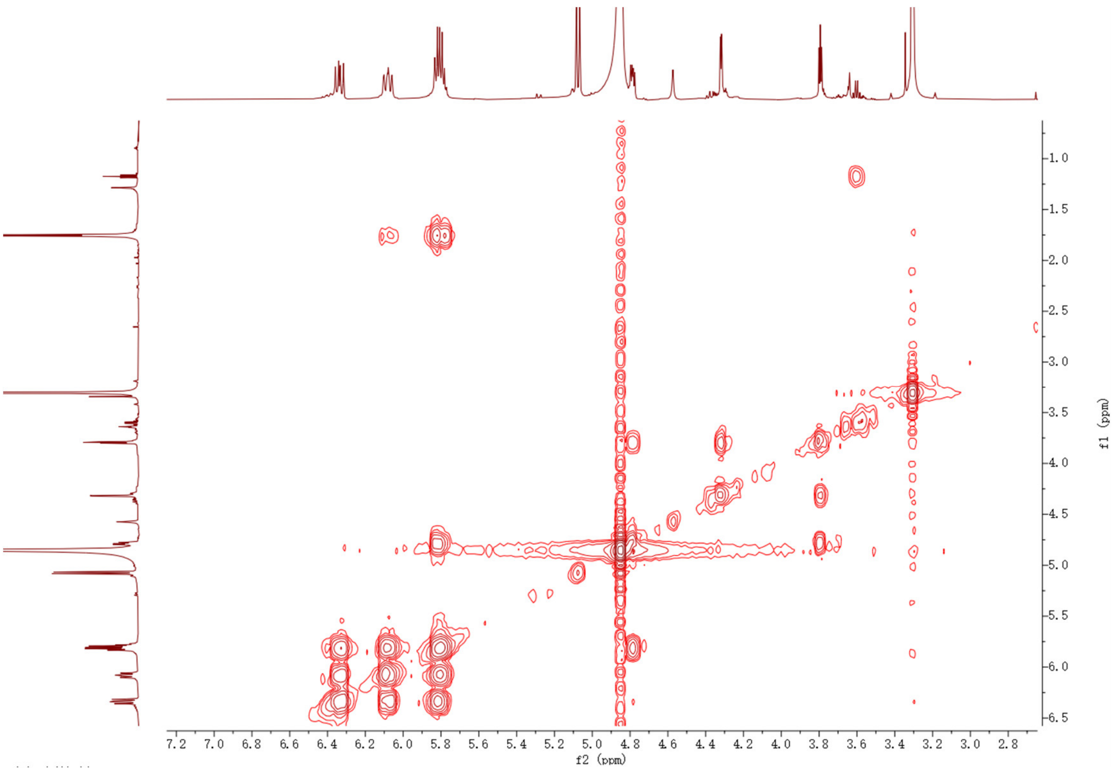

Figure S13. ^{1}H-^{1}H **COSY of compound 3 in** CD_3OD**.**

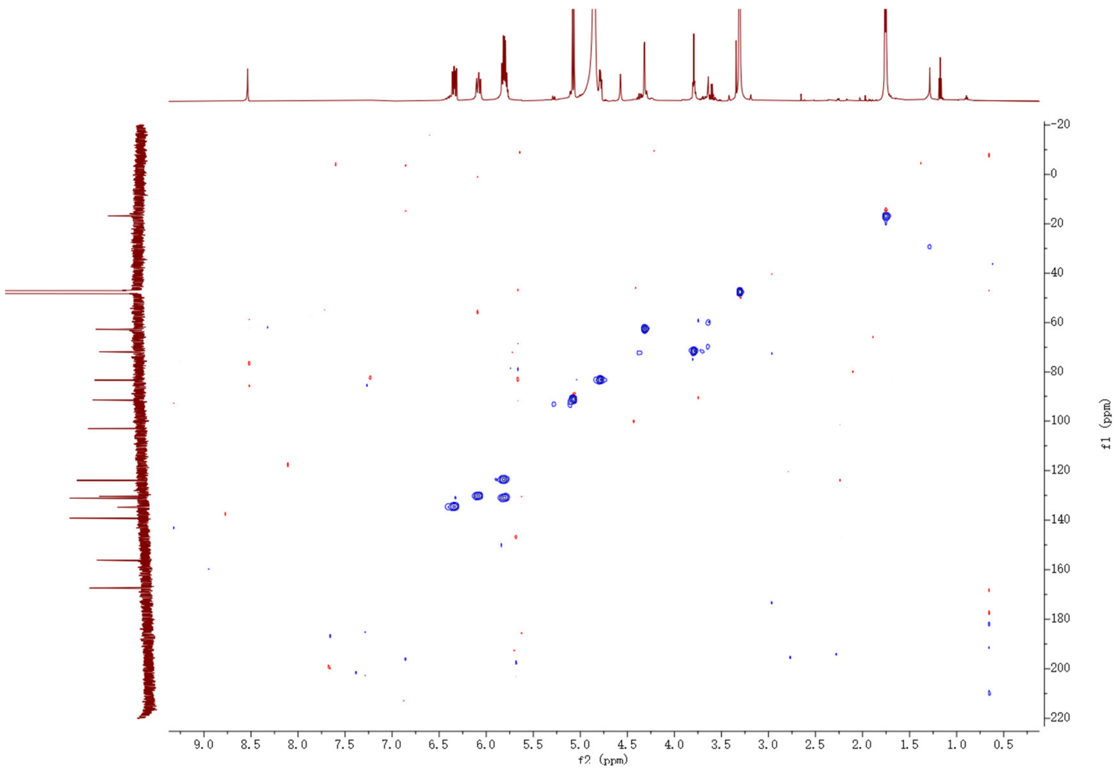

Figure S14. HSQC of compound 3 in CD_3OD.

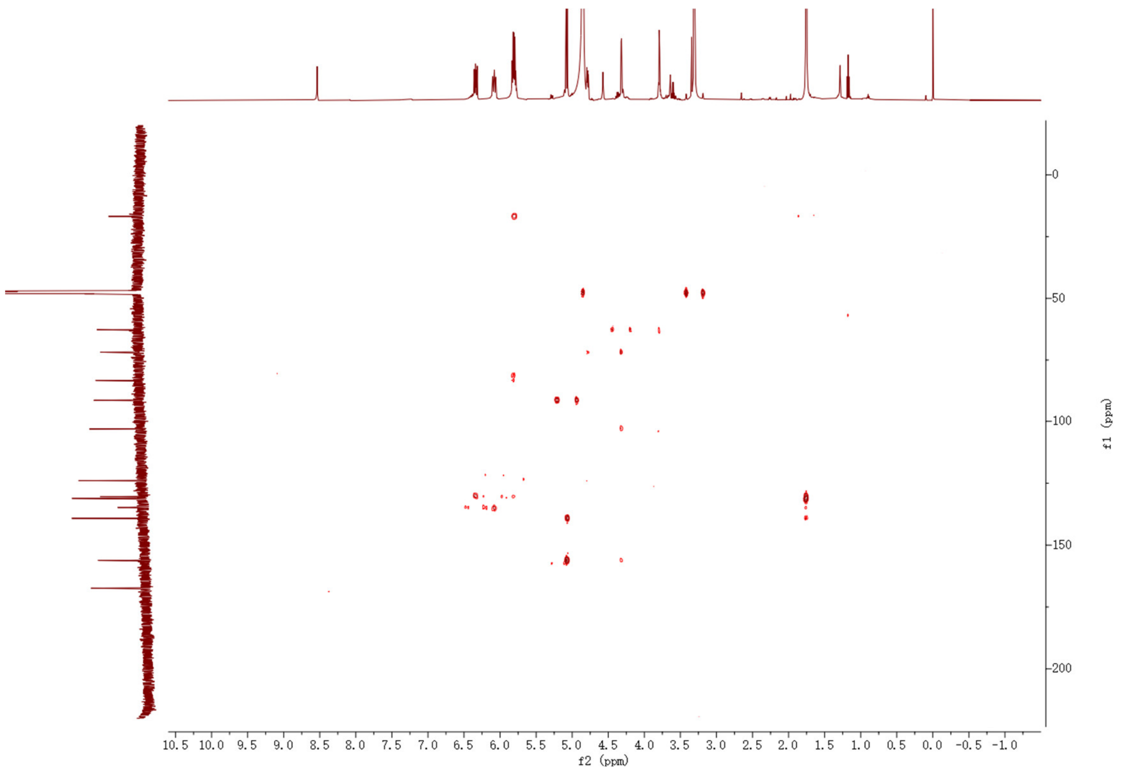

Figure S15. HMBC of compound 3 in CD_3OD.

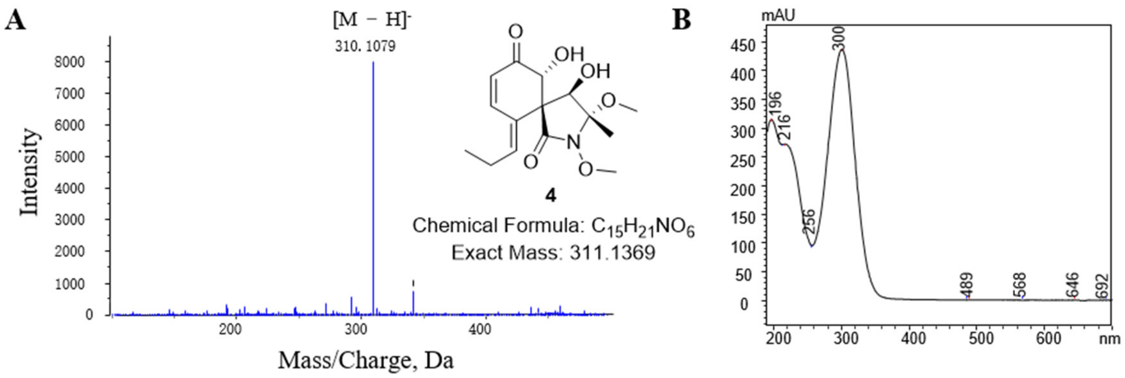

Figure S16. HRMS and UV spectra of compound 4.

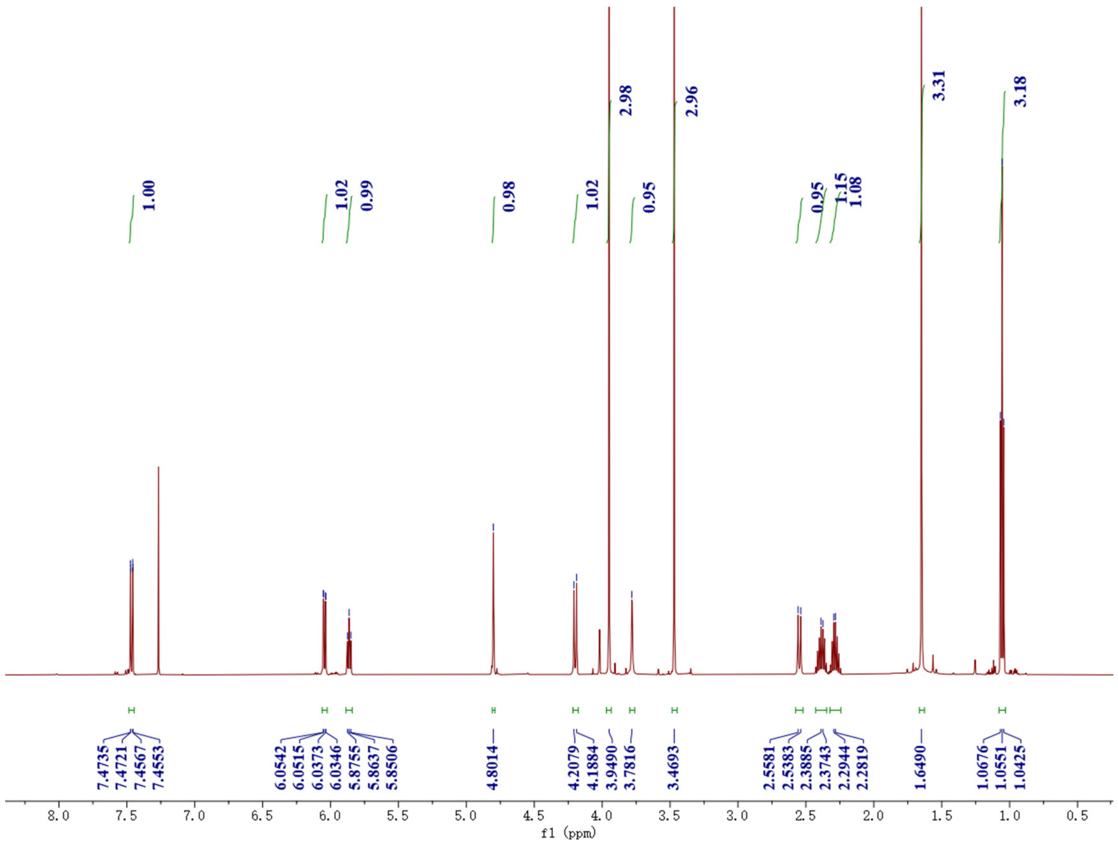

Figure S17. ^{1}H NMR of compound 4 in $CDCl_3$ at 600 MHz.

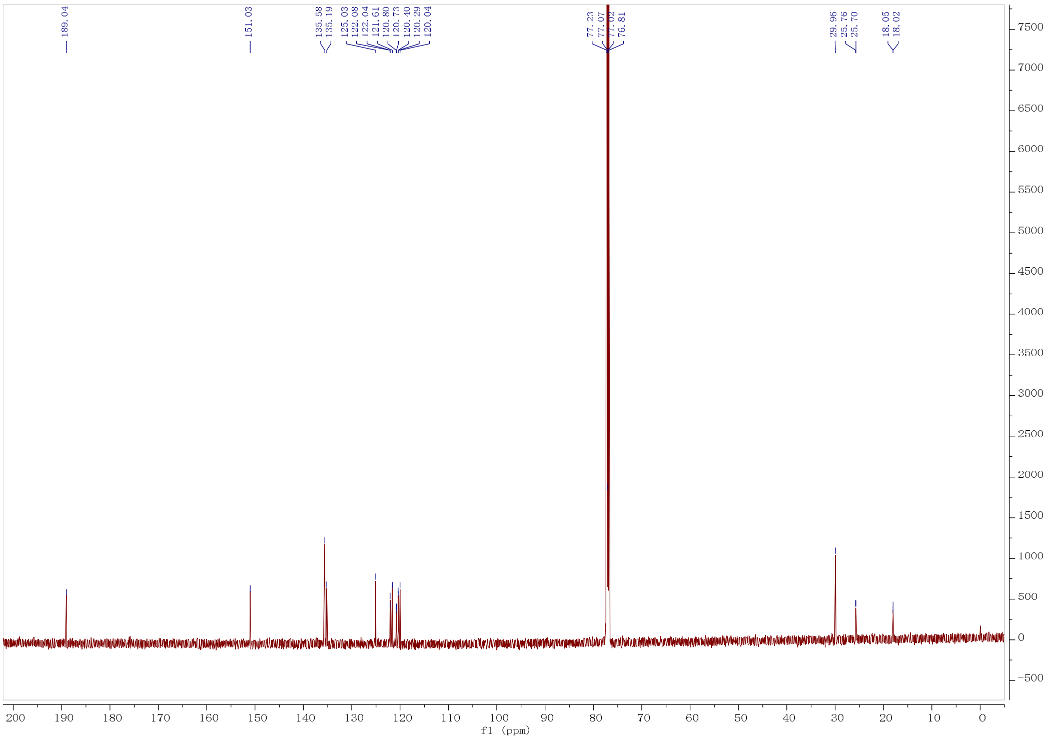

Figure S18. ^{13}C NMR of compound 4 in $CDCl_3$ at 150 MHz.

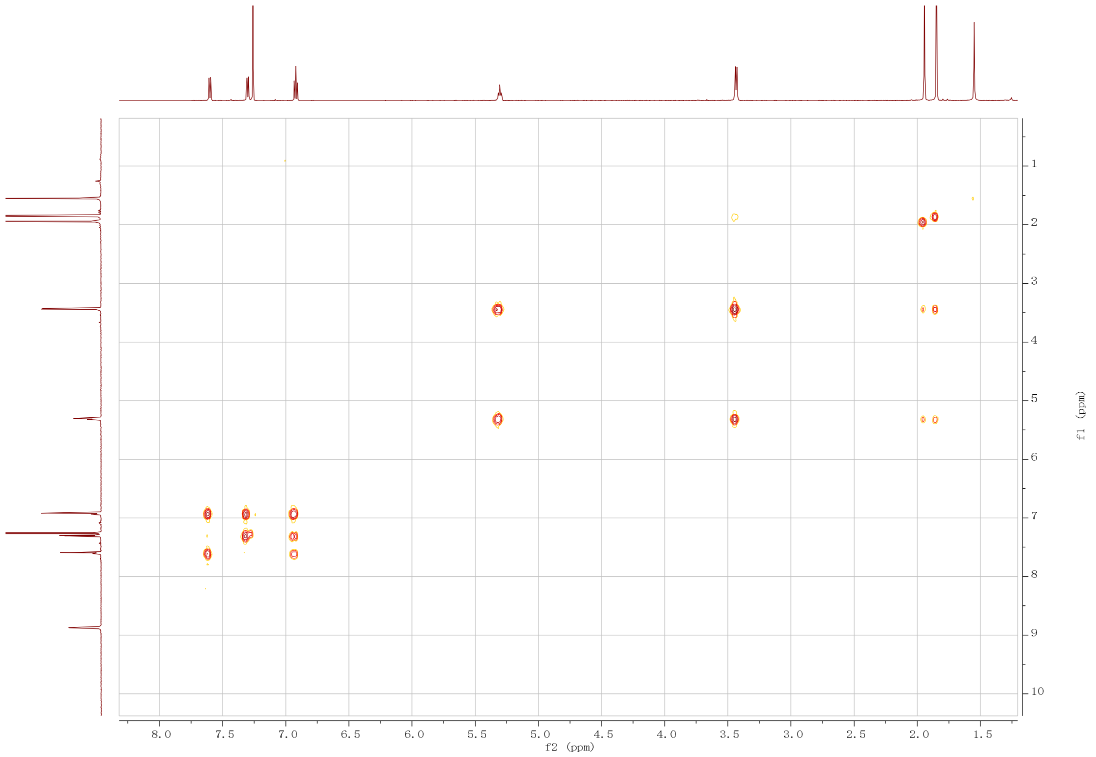

Figure S19. ^1H-^1H **COSY of compound 4 in** $CDCl_3$.

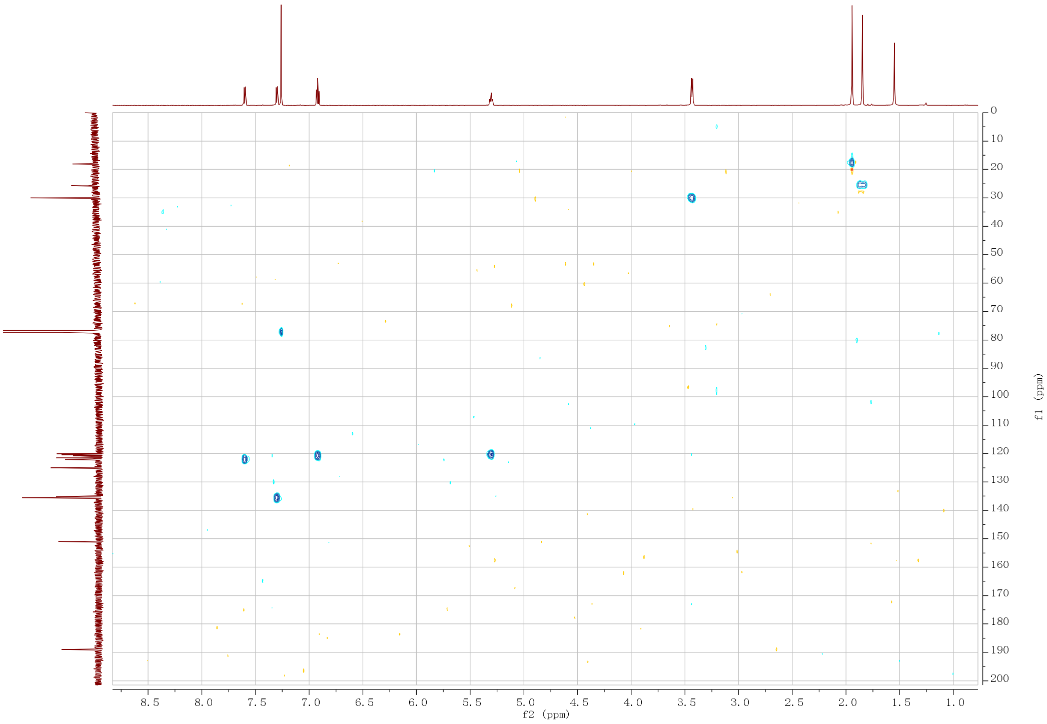

Figure S20. HSQC of compound 4 in $CDCl_3$.

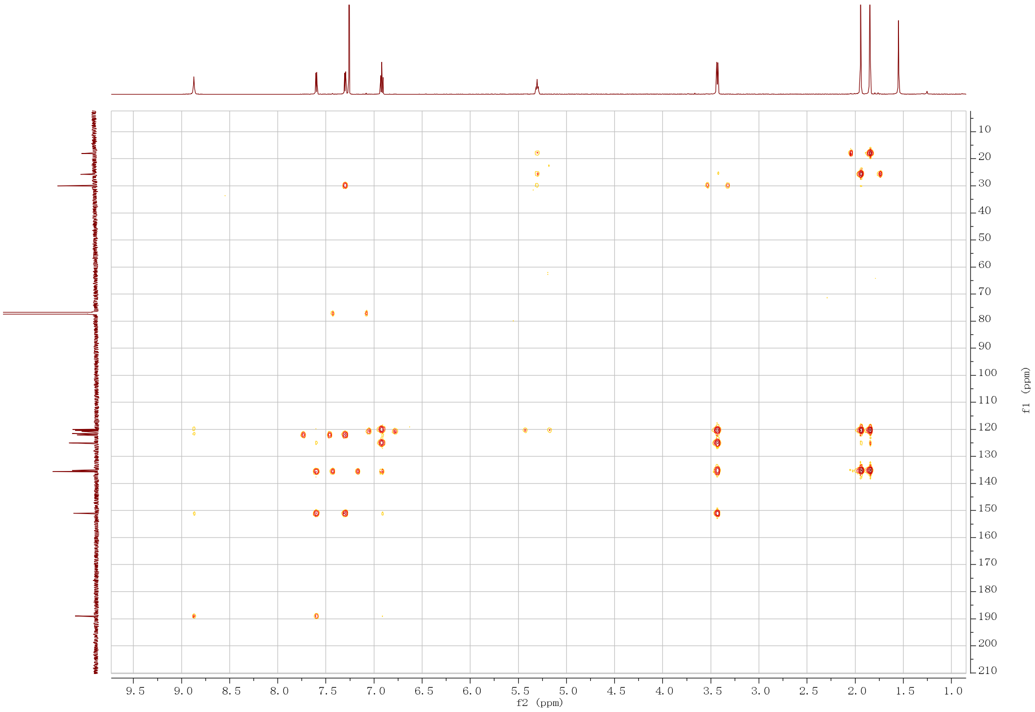

Figure S21. HMBC of compound 4 in $CDCl_3$.

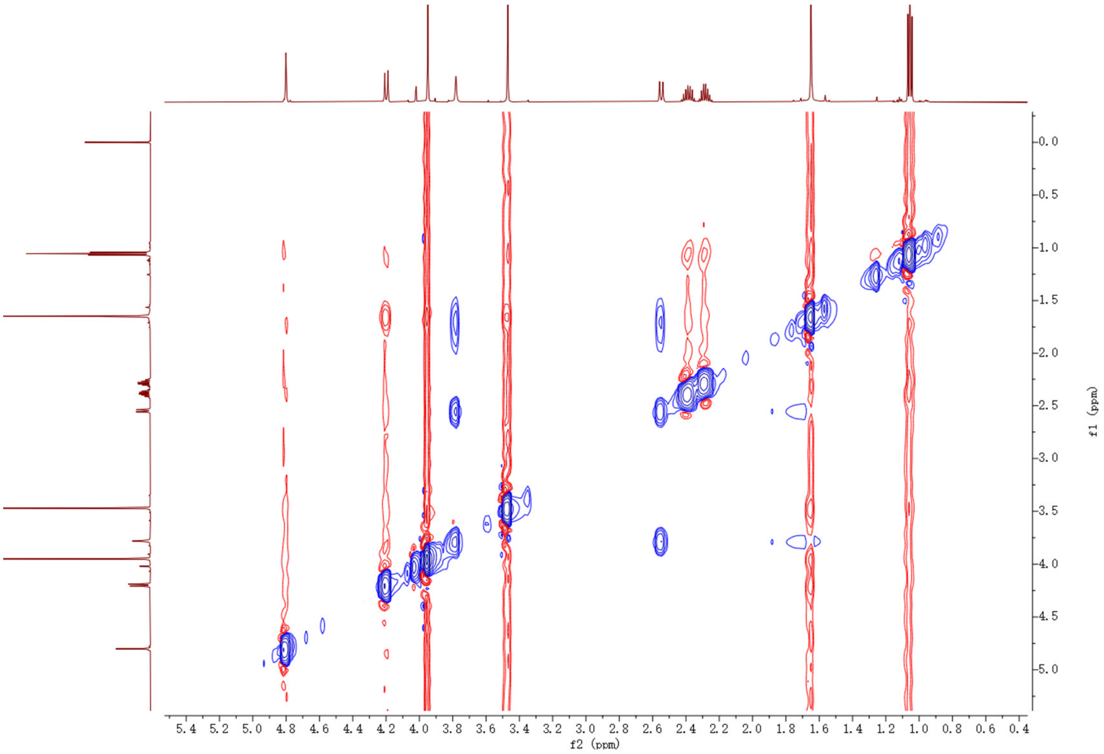

Figure S22. NOESY of compound 4 in $CDCl_3$.

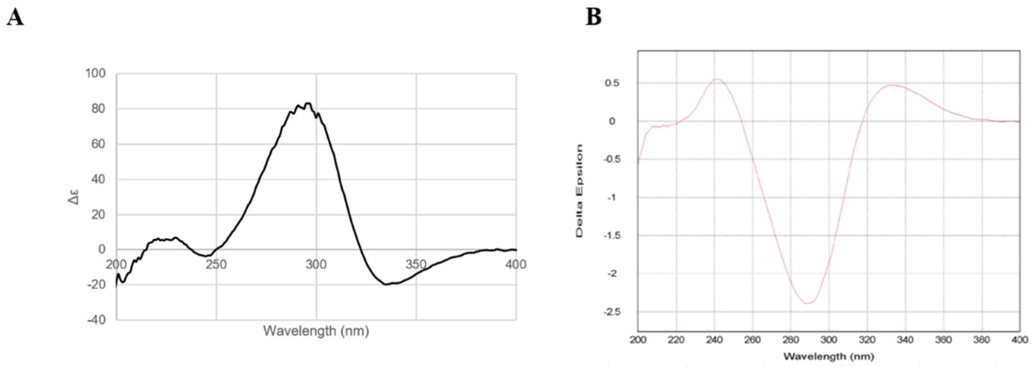

Figure S23. Comparative analysis of CD spectra between compound 2 and spirostaphylotrichin X

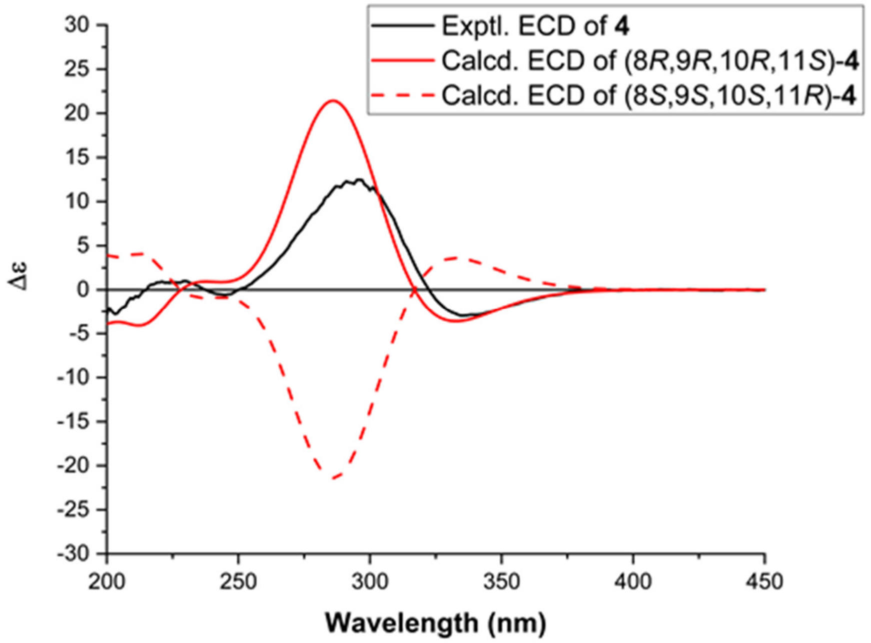

Figure S24. Experimental ECD curves of 4 (solid black line), M062X/TZVP//B3LYP/6-31G(d) calculated ECD spectra of (8*R*,9*R*,10*R*,11*S*)-4 (solid red line) and (8*S*,9*S*,10*S*,11*R*)-4 (dash red line).

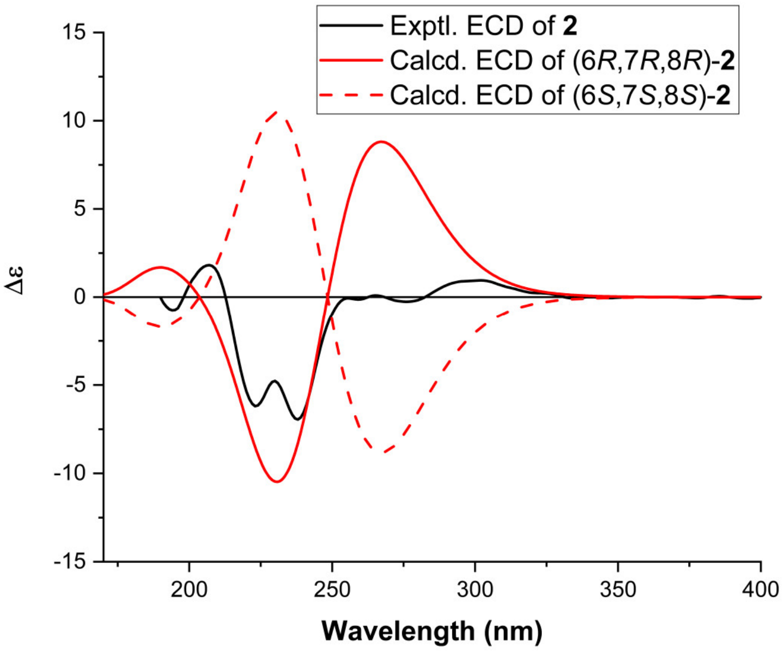

Figure S25. Experimental ECD curves of 2 (solid black line), M062X/TZVP//B3LYP/6-31G(d) calculated ECD spectra of (6*R*,7*R*,8*R*)-2 (solid red line) and (6*S*,7*S*,8*S*)-2 (dash red line).

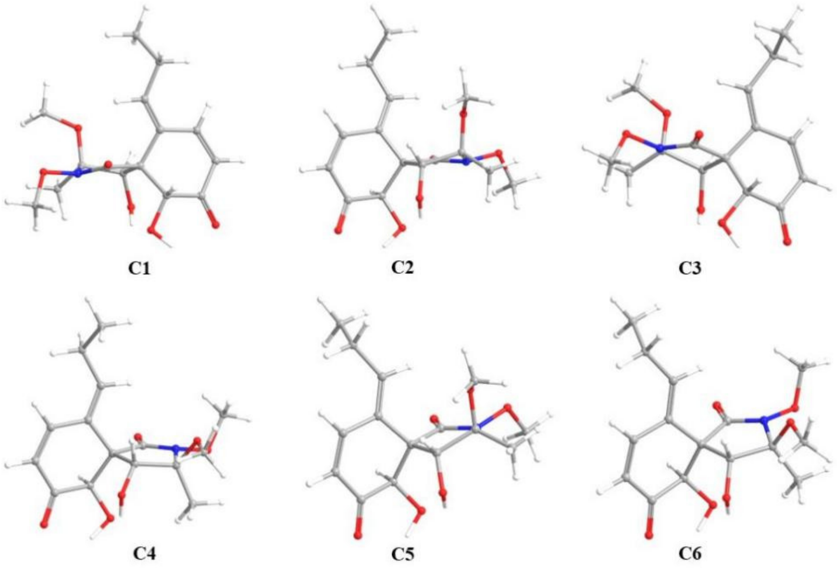

Figure S26. B3LYP/6-31G(d) optimized lowest energy conformers for (8*R*,9*R*,10*R*,11*S*)-4.

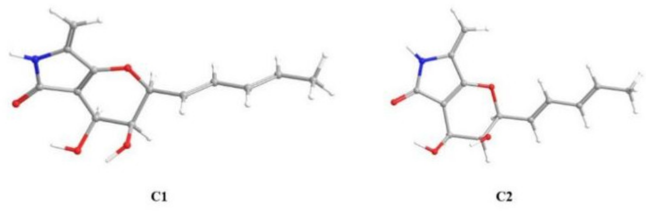

Figure S27. B3LYP/6-31G(d) optimized lowest energy conformers for (6*S*,7*S*,8*S*)-2.

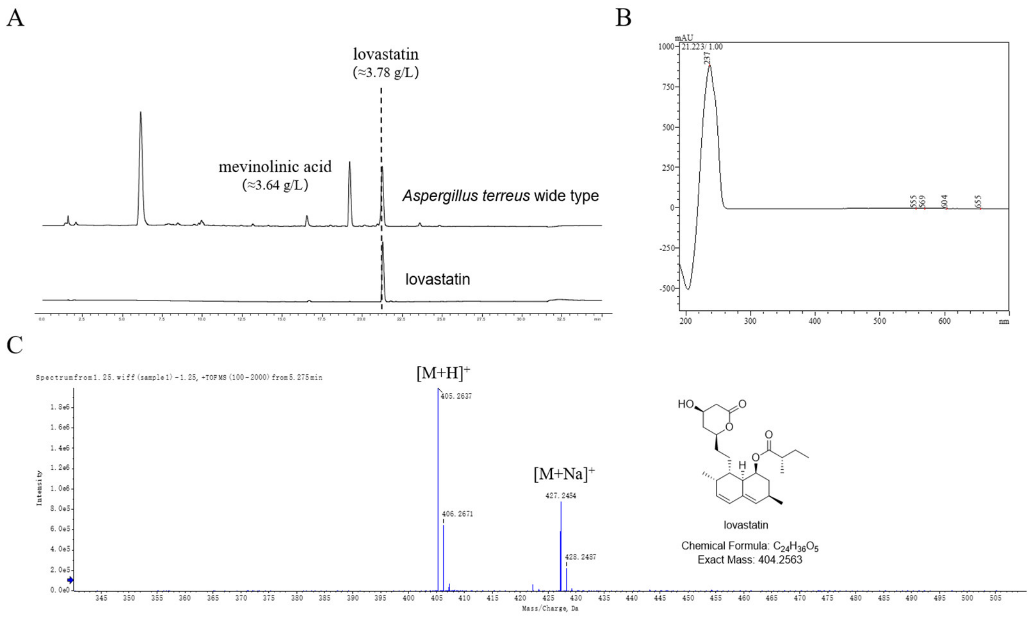

Figure S28. HPLC analysis of *A. terreus* and identification of lovastatin.

Supplementary References

Abraham, W.R.; Meyer, H.; Abate, D. Curvupallides, a new class of alkaloids from the fungus *Curvularia pallescens*. *Tetrahedron* **1995**, *51*, 4947-4952.

Cao, F.; Cheng, J.T.; Chen, X.A.; Li, Y.Q.; Mao, X.M. Development of an efficient genetic system in a gene cluster-rich endophytic fungus *Calcarisporium arbuscula* NRRL 3705. *J. Microbiol. Methods* **2018**, *151*, 1-6.

Cao, F.; Ma, L.F.; Hu, L.S.; Xu, C.X.; Chen, X.; Zhan, Z.J.; Zhao, Q.W.; Mao, X.M. Coordination of polyketide release and multiple detoxification pathways for tolerable production of fungal mycotoxins. *Angew. Chem. Int. Ed.* **2023**, *62*, e202214814.

Chiang, Y.M.; Lin, T.S.; Chang, S.L.; Ahn, G.; Wang, C. C. C. An *Aspergillus nidulans* platform for the complete cluster refactoring and total biosynthesis of fungal natural products. *ACS Synth. Biol.* **2021**, *10*, 173-182.

Mao, X.M.; Zhan, Z.J.; Grayson, M. N.; Tang, M.C.; Xu, W.; Li, Y.Q.; Yin, W.B.; Lin, H.C.; Chooi, Y.H.; Houk, K. N.; et al. Efficient biosynthesis of fungal polyketides containing the dioxabicyclo-octane ring system. *J. Am. Chem. Soc.* **2015**, *137*, 11904-11907.

Nguyen, K. T.; Ho, Q. N.; Pham, T. H.; Phan, T.N.; Tran, V.T. The construction and use of versatile binary vectors carrying *pyrG* auxotrophic marker and fluorescent reporter genes for *Agrobacterium*-mediated transformation of *Aspergillus oryzae*. *World J. Microbiol. Biotechnol.* **2016**, *32*, 204.

Stephens, P. J.; Harada, N. ECD Cotton effect approximated by the Gaussian curve and other methods. Chirality **2010**, *22*, 229-233.

Wang, J.; Chen, F.; Liu, Y.; Liu, Y.; Li, K.; Yang, X.; Liu, S.; Zhou, X.; Yang, J. Spirostaphylotrichin X from a marine-derived fungus as an anti-influenza agent targeting RNA polymerase PB2. *J. Nat. Prod.* **2018**, *81*, 2722-2730.
